# Autism spectrum disorder profiles in RASopathies: A systematic review

**DOI:** 10.1002/mgg3.2428

**Published:** 2024-04-05

**Authors:** Edward Debbaut, Jean Steyaert, Mouna El Bakkali

**Affiliations:** ^1^ Center for Developmental Psychiatry, Department of Neurosciences KU Leuven Leuven Belgium; ^2^ Leuven Autism Research (LAuRes) KU Leuven Leuven Belgium; ^3^ Faculty of Medicine KU Leuven Leuven Belgium

**Keywords:** autism spectrum disorder, cardio‐facio‐cutaneous syndrome, Costello syndrome, developmental phenotype, neurofibromatosis type 1, Noonan syndrome, RASopathies

## Abstract

**Background:**

RASopathies are associated with an increased risk of autism spectrum disorder (ASD). For neurofibromatosis type 1 (NF1) there is ample evidence for this increased risk, while for other RASopathies this association has been studied less. No specific ASD profile has been delineated so far for RASopathies or a specific RASopathy individually.

**Methods:**

We conducted a systematic review to investigate whether a specific RASopathy is associated with a specific ASD profile, or if RASopathies altogether have a distinct ASD profile compared to idiopathic ASD (iASD). We searched PubMed, Web of Science, and Open Grey for data about ASD features in RASopathies and potential modifiers.

**Results:**

We included 41 articles on ASD features in NF1, Noonan syndrome (NS), Costello syndrome (CS), and cardio‐facio‐cutaneous syndrome (CFC). Individuals with NF1, NS, CS, and CFC on average have higher ASD symptomatology than healthy controls and unaffected siblings, though less than people with iASD. There is insufficient evidence for a distinct ASD phenotype in RASopathies compared to iASD or when RASopathies are compared with each other. We identified several potentially modifying factors of ASD symptoms in RASopathies.

**Conclusions:**

Our systematic review found no convincing evidence for a specific ASD profile in RASopathies compared to iASD, or in a specific RASopathy compared to other RASopathies. However, we identified important limitations in the research literature which may also account for this result. These limitations are discussed and recommendations for future research are formulated.

## Graphical abstract

ASD symptom profiles in RASopathies were reviewed and compared. RASopathies show an unimodal, continuously distributed increase in ASD symptoms compared to the general population. Differences in the amount of ASD symptoms exist between RASopathies, but no specific symptom profile emerges. Effects of age, sex, cognition, ADHD, and genotype are discussed.

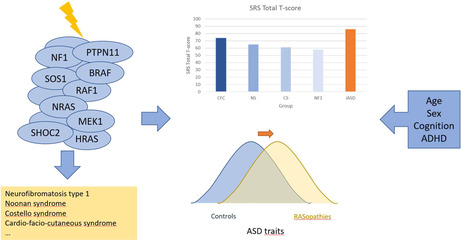

## 1 INTRODUCTION

RASopathies are a group of disorders resulting from germline pathogenic variants in genes encoding components or regulators of the Ras/mitogen‐activated protein (MAP) kinase signaling pathway (Rauen, 2022). The Ras/MAP‐kinase pathway connects cell surface receptors to transcription factors regulating gene expression of proteins important for cell survival, differentiation, and replication. Hyperactivation of the Ras/MAP‐kinase pathway leads to abnormal tissue growth. The most prevalent and best‐described RASopathies are neurofibromatosis type 1 (NF1), Noonan syndrome (NS), Costello syndrome (CS), and cardio‐facio‐cutaneous syndrome (CFC). The common pathogenesis explains the overlap in phenotypic features such as craniofacial dysmorphia, cardiac malformations, increased tumor risk, cutaneous manifestations, cognitive deficits, and psychiatric morbidity (Alfieri et al., 2014; Bessis et al., 2019; Friedman et al., 2002; Jafry & Sidbury, 2020; McCubrey et al., 2007; Rauen et al., 2018; Siegel et al., 2011, 2012; Tidyman & Rauen, 2016). The latter includes autism spectrum disorder (ASD) (Adviento et al., 2014; Chisholm et al., 2018; Garg et al., 2017). The Diagnostic and Statistical Manual of Mental Disorders, fifth edition (DSM‐5) defines ASD by two symptom criteria, each having its own sub‐criteria (American Psychiatric Association, 2013). Criterion A requires persistent deficits in social communication and social interaction and criterion B requires restricted and repetitive patterns of behavior and interests (RRB). As various combinations of sub criteria to fulfill criterion B, different symptoms to fulfill the sub‐criteria and different degrees of severity of the symptoms are possible, ASD is a heterogeneous disorder with a variable behavioral presentation. Challenges arise in the evaluation of ASD traits in RASopathies due to psychiatric comorbidity (Garg, Lehtonen, et al., 2013). Different instruments exist to describe ASD traits. Some instruments such as the Social Communication Questionnaire (SCQ) (Berument et al., 1999), the Modified Checklist for Autism in Toddlers (M‐CHAT) (Robins et al., 2001), and the Social Responsiveness Scale (SRS) (Constantino, 2002) allow to quantify the amount of ASD traits and screening for ASD. Others such as the Autism Diagnostic Interview‐Revised (ADI‐R) (Rutter et al., 2003) and the Autism Diagnostic Observation Schedule (ADOS) (Lord et al., 2012) are used for an elaborate diagnostic assessment and yield a classification. No instrument matches the clinical diagnosis of ASD perfectly. One recent review and meta‐analysis portrayed the ASD characteristics in NF1 (Chisholm et al., 2018a). To our knowledge, no review exists reviewing different RASopathies and comparing the ASD profiles across these different syndromes.

Delineating a particular profile of ASD symptoms in RASopathies compared to each other or compared to idiopathic ASD (iASD) may point to a set of symptoms being associated with the RAS/MAP‐kinase pathway and could lead to a better understanding of the RAS/MAPkinase pathway as a molecular mechanism of ASD. In clinical practice, this could also lead to a better detection of ASD symptoms and allocation of intervention strategies, as this may diverge from the approach in the general population. Therefore, our main research questions are, firstly, whether existing research shows the ASD symptom profile in RASopathies is different from that in iASD and secondly, whether the ASD profile is different in individual RASopathies.

## 2 MATERIALS AND METHODS

### 2.1 Search method and eligibility criteria

We conducted a systematic search following the preferred reporting items for systematic reviews and meta‐analyses (PRISMA) updated guidelines (Page et al., 2021) in MEDLINE (PubMed), Web of Science, and Open Grey on November 28, 2023. The search process was examined and reported as recommended by the search extension to the PRISMA statement (PRISMA‐S) (Rethlefsen et al., 2021). The search sequences used in MEDLINE, Web of Science, and Open Grey can be found in Supplement S1.

Inclusion criteria were: (1) original studies, (2) involving human participants, (3) reporting about participants with a clinical or molecular diagnosis of a RASopathy, (4) using an instrument measuring observable ASD traits or ASD‐related behavior, (5) published in English, (6) published after January 1, 1994.

Exclusion criteria were: (1) case reports and case series, due to selection bias risk, (2) articles only reporting non‐behavioral data (e.g., stress physiology, biochemistry, electro‐encephalogram) or performance on explicit tasks of social processing without considering DSM‐5 ASD characteristics, (3) articles only reporting ASD diagnoses without specifying a measuring instrument or fulfillment of diagnostic sub criteria, (4) completely overlapping samples with no additional relevant analysis, (5) articles only reporting data from measuring instruments that are not used by any other study and, moreover, (a) do not make a comparison between different RASopathies or between a RASopathy and idiopathic ASD, or (b) do not analyze the data in function of any of our prespecified possible moderating factors: age, sex, ADHD, cognition and genotype. This is because in these cases a relevant and reliable interpretation of the data in the context of our research questions is not possible.

The time of publication was limited to 1994–2023, as the Diagnostic and Statistical Manual of Mental Disorders, fourth edition (DSM‐IV) was published in 1994 (American Psychiatric Association, 1994). The DSM‐IV did not use the term ASD but bundled a group of different syndromes in the chapter Pervasive developmental disorders (PDD), including Autistic Disorder, Asperger's Disorder, and Pervasive Developmental Disorder—Not Otherwise Specified (PDD‐NOS). Although the exact DSM‐IV criteria for these syndromes differed from the DSM‐5 criteria for ASD, research showed that the outer limits of the population with PDD and ASD were not substantially different. In addition, even during the DSM‐IV era the term ASD was already used much more than PDD in the scientific literature (King et al., 2014).

We chose not to exclude articles based on absent or incomplete molecular confirmation of the RASopathy diagnoses. The most important reason for this decision was that RASopathies had already been described clinically before the genes involved in the RAS/MAPkinase pathway and RASopathies were identified. Hence, excluding publications because of incomplete or absent molecular confirmation could have excluded relevant research findings. Including genes nomenclature as search terms could have been an alternative approach for the literature search, but in our paper we chose to approach the research questions through the lens of clinically recognizable syndromes, as is still common practice in clinical genetics. Since different RASopathy mutations have been described in molecular terms, it is recommended to use these in publications on original research, whenever possible. However, the aim of our review was to review the available literature that potentially contains an answer to the research questions, and not to report original molecular research. Consequently, the terms and definitions most commonly used by authors of the reviewed literature were the starting point of the review. Furthermore, concepts such as “RASopathy” and “RAS/MAPkinase” were included in our search terms, making it very unlikely that publications on the clinical phenotype related to a mutation in the RAS/MAPkinase pathway without applying a syndrome name to it would not be picked up by our search.

We included data on RASopathy subjects, regardless of ASD diagnosis. This was motivated by a number of considerations. In our research question, we refer to the ASD symptom profile as the behaviors that are described by the DSM‐5 criteria for ASD without necessarily completely fulfilling the DSM‐5 criteria. In our introduction, we also note that insight into the ASD profile in RASopathies may indicate that the RAS/MAPkinase pathway is associated with specific ASD symptoms, which may lead to a better understanding of the association between genetic factors and behavioral symptoms. Naturally, to achieve this, we must also take into account RASopathy patients who do not have ASD but may exhibit characteristics of ASD to a greater or lesser extent. Furthermore, there is no uniform way in which the ASD classification is assigned in the research literature, making the delineation of RASopathy+ASD as a study population ambiguous. In addition, commonly used measuring instruments (such as the SRS) provide quantitative data that are continuously distributed, not only within groups with and groups without ASD, but also between ASD and non‐ASD populations.

Records were first screened by title and abstract by one reviewer (M.E.B.). If the record was possibly eligible after title and abstract review, the full text was reviewed. Afterward, the same search and selection process was carried out independently by another reviewer (E.D.) and compared to the results by M.E.B. In case of disagreement consensus between the two authors was reached.

### 2.2 Data extraction

Data extracted from the selected articles were: first author, year of publication, study design, specific RASopathy/RASopathies, control group, number and age range of participants, ASD‐related outcomes, the relationship of other prespecified outcome data (age, sex, ADHD, cognition and genotype) to ASD‐related outcomes, molecular confirmation, and geographical location. Because all but one of the included studies were cross‐sectional, quality was rated according to the Strengthening the Reporting of Observational Studies in Epidemiology (STROBE) recommendations, using the 22‐item checklist for cross‐sectional studies (Vandenbroucke et al., 2007; Von Elm et al., 2007).

## 3 RESULTS

### 3.1 Included studies

**FIGURE 1:**
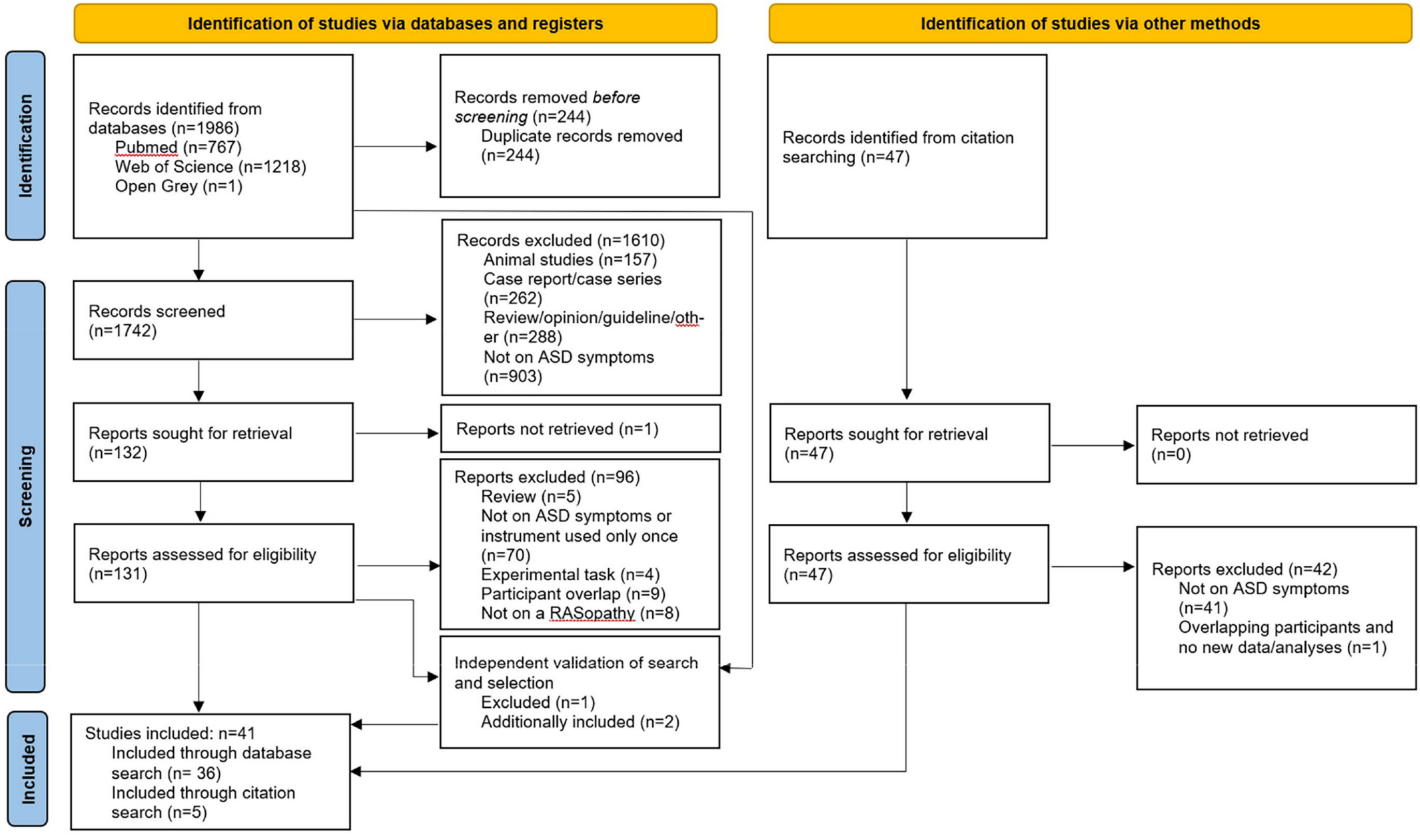
PRISMA flowchart. Flowchart of the search strategy, and in‐ and exclusion of articles following the updated PRISMA guidelines (Rethlefsen et al., 2021).

The selection process resulted in the inclusion of 41 articles and is visualized as a PRISMA flowchart in Figure 1 (Page et al., 2021). It is further described in Supplement S1.

### 3.2 Characteristics of the included studies

Table S1 (Supplement S2) summarizes the characteristics of the included studies. Because there was clear evidence of overlap between the samples of the different included studies, we attempted to estimate the number of unique participants. More information about these calculations can be found in Supplement S3. We estimated that the included studies together included approximately 2601 unique participants with NF1 (82.5% of the total amount of unique RASopathy participants included in our review), 313 with NS (9.9%), 101 with CS (3.2%), 126 with CFC (4%), 4 with Noonan‐like syndrome with loose anagen hair (0.1%) and 6 with NS with multiple lentigines (0.2%). These figures must be interpreted with caution because the overlap between the different samples could not always be excluded or confirmed with complete certainty. At the same time, they are certainly more accurate than a simple sum of the different samples of the individual studies and they show that NF1 participants are overrepresented in comparison to the other RASopathies.

There were six studies (14.6%) comparing two or more RASopathies directly, while 15 studies (36.6%) compared one or more RASopathies to a control sample, which could be unaffected siblings (US), healthy controls from the general population (HC), a non‐RASopathy ASD group, or another non‐RASopathy comparison group. Seven studies (17.1%) used a HC comparison group, two studies (4.8%) compared with US, and seven studies (17.1%) compared with an ASD group. Six studies (14.6%) had molecular confirmation of the diagnosis in all participants with a RASopathy. Seven studies (17.1%) had confirmation in a fraction of participants, and 28 studies (68.3%) did not report molecular confirmation. We did not analyze data of individuals with Noonan‐like syndrome with loose anagen hair and NS with multiple lentigines, as there was not enough data to draw conclusions. Rating of the quality of the articles according to the STROBE recommendations is summarized in Table S2 (Supplement S2).

## 4 RESULTS

Table S3 (Supplement S2) summarizes the outcomes of the included studies.

### 4.1 ASD features in general

#### 4.1.1 Screening instruments

##### 4.1.1.1 Social skills improvement system (SSIS)

SSIS data provide evidence for weaker social skills in NF1 and NS compared to the general population, but no significant differences between both RASopathies. In one study 44% of NF1 participants (*n* = 39), 41% of NS participants (*n* = 39), and 13% of US (*n* = 32) scored lower than one standard deviation (SD) below the normative mean on the SSIS Social skills subscale, indicating low social skills (Pierpont et al., 2018). 5% of NF1 and 15% of NS participants scored even lower than two SD below the normative mean, while none of the US group did. Mean NF1 and NS scores did not differ significantly, but were significantly lower than US scores. Payne et al. used the same measure in a sample of children with NF1 (*n* = 122) and demonstrated the mean score to be 0.65 SD lower than the normative mean, with 36.9% of participants scoring lower than one SD, 24.6% scoring lower than 1.5 SD, and 8.2% scoring lower than 2.5 SD below the mean (Payne et al., 2020).

Glad et al. found 32% and 24% of their NF1 sample scoring one SD below the normative mean, depending on whether data from respectively early childhood or school age were analyzed (Glad et al., 2021). However, scores lower than 2 SD below the mean were found in only 4% in early childhood and 8% at school age.

##### 4.1.1.2 Social communication questionnaire (SCQ), modified checklist for autism in toddlers (M‐CHAT) and childhood autism Spectrum test (CAST)

The SCQ, M‐CHAT, and CAST were often administered within the same study, the SCQ or CAST being used for participants with a higher chronological or mental age, and the M‐CHAT for younger participants. All instruments yield a score but also have a screening cut‐off. Put together, these studies indicate an elevated rate of ASD symptoms in RASopathies, with quantitative differences between the RASopathies: the highest amount of symptoms in CFC, the lowest in NF1, and CS and NS showing an intermediary level of ASD symptoms.

**FIGURE 2:**
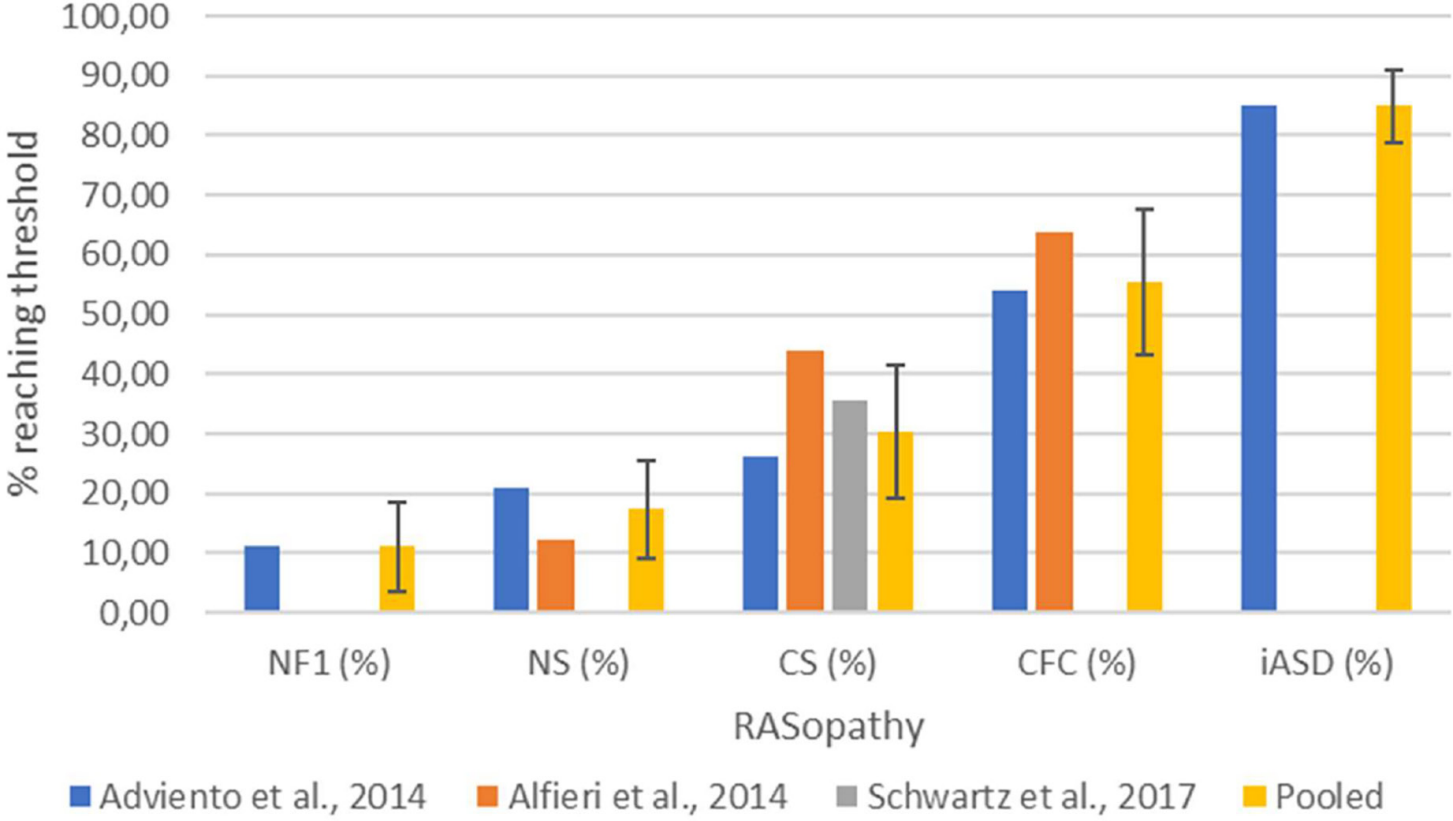
Proportion of participants reaching the SCQ or M‐CHAT threshold. Error bars represent 95% confidence intervals. Separate data from Adviento et al. (2014), Alfieri et al. (2014), and Schwartz et al. (2017) are displayed per RASopathy and for iASD, as well as the value obtained by pooling data from these studies whenever possible. CFC, cardio‐facio‐cutaneous syndrome; CS, Costello syndrome; M‐CHAT, modified checklist for autism in toddlers; NF1, neurofibromatosis type 1; NS, Noonan syndrome; SCQ, social communication questionnaire.

Adviento et al. compared SCQ data from NF1 (*n* = 66), NS (*n* = 48), CS (*n* = 43), CFC (*n* = 54), US (*n* = 117) and iASD (*n* = 133) (Adviento et al., 2014). No US participants reached the cut‐off, whereas 85%, 54%, 26%, 21% and 11% of iASD, CFC, CS, NS, and NF1 subjects did, respectively. Alfieri et al. screened NS (*n* = 33), CS (*n* = 9), and CFC (*n* = 11) patients using the SCQ or the M‐CHAT (Alfieri et al., 2014). CFC showed the highest proportion of participants reaching the cut‐off (64%), while 44% and 12% of participants with CS and NS also reached the cut‐off, respectively (Figure 2). These differences in proportions were all statistically significant. Tinker et al. screened NF1 children using the M‐CHAT and CAST (Tinker et al., 2014). Of the children for whom the questionnaire had completely been filled out, none screened positive on the M‐CHAT (0/20), while on the CAST 12.5% (5/40) screened positive. Both proportions were not significantly different from positive screening rates in the general population, but CAST mean and median scores were higher than reported from a control population. Schwartz et al. studied CS (*n* = 14) using the SCQ and the M‐CHAT (Schwartz et al., 2017). All the participants screening positive were in the younger age group, screened using the M‐CHAT (*n* = 7). Depending on the cut‐off used they found that 36% (5 out of 14) or 14% (2 out of 14) of their participants screened positive.

Pooling data from the studies mentioned above on the proportions in each RASopathy exceeding the SCQ/M‐CHAT cut‐off, and calculating 95% confidence intervals (95%CI) based on a binomial distribution, we found that 11% of NF1 participants (95%CI 3.45;18.55) screened positive. Figures in NS (17.28%, 95%CI 9.05;25.52), CS (30.30%, 95%CI 19.22;41.39), and especially CFC (55.38%, 95%CI 43.30;67.47) were much higher. The proportion exceeding the cut‐off in CFC was significantly higher than in the other RASopathies (two‐tailed *α* < 0.05). Furthermore, the proportion exceeding the cut‐off in CS was significantly higher than in NF1. Percentages of participants exceeding the SCQ/M‐CHAT cut‐off in RASopathies across studies, including the result of pooling these data, are displayed in Figure 2.

##### 4.1.1.3 Social responsiveness scale (SRS)

**FIGURE 3:**
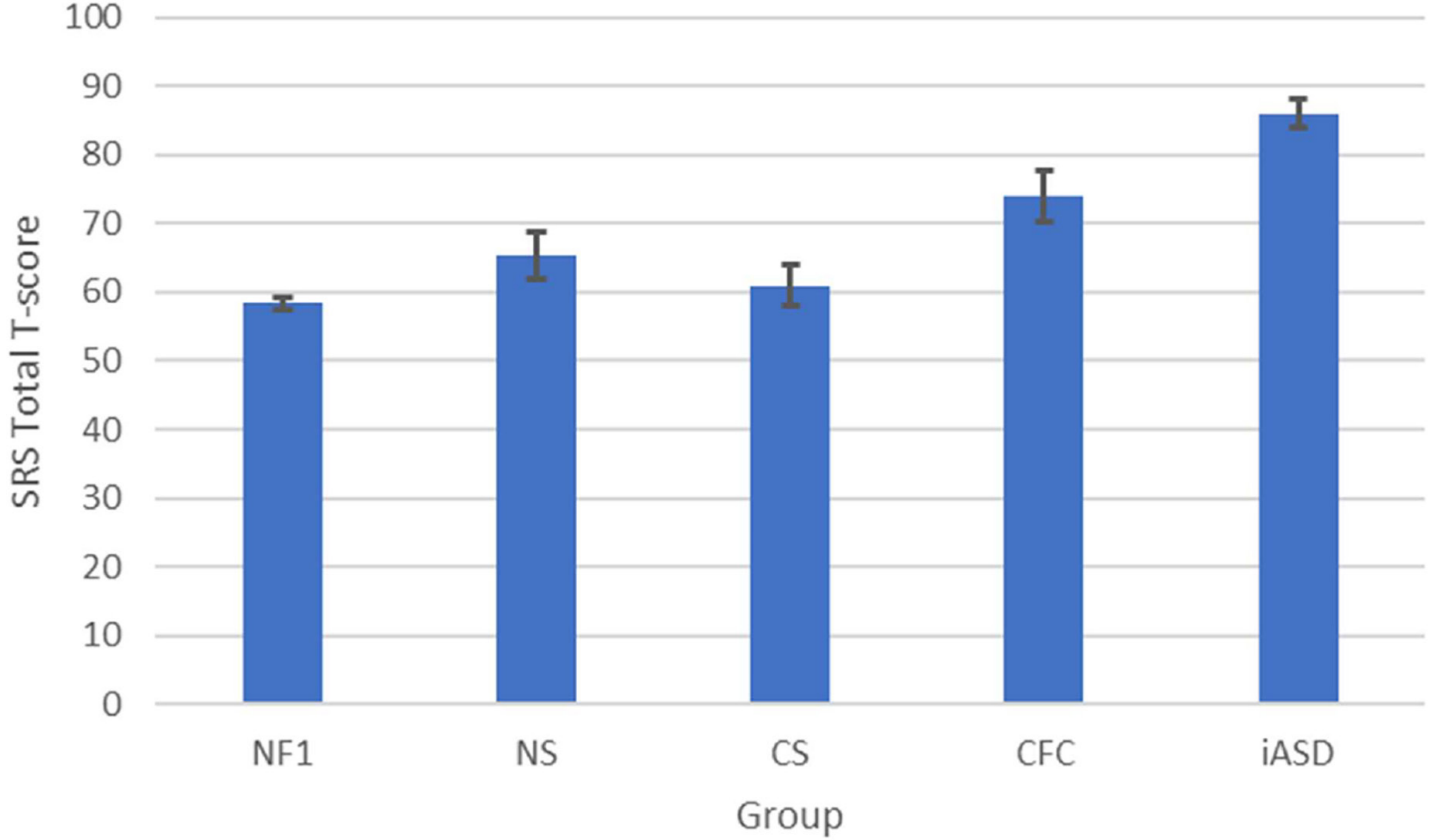
SRS total *T* scores in different RASopathies and iASD comparison group. Error bars represent 95% confidence intervals. The scores for CFC, CS, and iASD have been adopted from Adviento et al. (2014). The score for NF1 has been calculated by pooling data from 8 studies (Chisholm et al., 2022; Huijbregts & de Sonneville, 2011; Lalancette et al., 2022; Lubbers et al., 2022; Morotti et al., 2021; Morris et al., 2016; Payne et al., 2020; van Eeghen et al., 2013). The score for NS has been calculated by pooling data from two studies (Adviento et al., 2014; Naylor et al., 2023). CFC, cardio‐facio‐cutaneous syndrome; CS, Costello syndrome; iASD, idiopathic autism spectrum disorder; NF1, neurofibromatosis type 1 regardless of ASD diagnosis; NF1+ASD, NF1 selected on ASD diagnosis; NS, Noonan syndrome; SRS, social responsiveness scale.

Like the SCQ, M‐CHAT, and CAST, the SRS yields a quantitative measure of ASD symptoms, but often a cut‐off is used (total *T* score ≥60 or ≥75) to classify individuals. Only one study reported SRS data on other RASopathies in addition to NF1, and again, CFC patients were found to have the highest level of ASD symptoms, and NF1 patients the lowest. Adviento et al. reported that NF1 (*n* = 78; mean 57), NS (*n* = 51; mean 65), CS (*n* = 40; mean 61) and CFC (*n* = 49; mean 74) had significantly higher SRS *T* scores than US (*n* = 114; mean 46), and significantly lower *T* scores than iASD (*n* = 167; mean 86). The mean CFC *T* score was significantly higher than in the other RASopathies (Adviento et al., 2014). One study in children with NF1 (*n* = 143) did not report mean scores but reported that 20.3% of their sample scored in the severe clinical range on the SRS (total *T* score ≥ 76) and 14.7% in the mild to moderate clinical range (59 < total *T* score < 76) (Hirabaru & Matsuo, 2018). Morris et al. pooled own and previously published SRS data in NF1 (*n* = 531) (Adviento et al., 2014; Constantino et al., 2015; Garg, Lehtonen, et al., 2013; Plasschaert et al., 2015; Walsh et al., 2013) and found total *T* scores to be continuously, unimodally distributed, the mean total *T* score (58.21) being pathologically shifted with 0.8 SD relative to norms from the general population. 13.2% of individuals scored in the severe clinical range and 26% in the mild to moderate clinical range (Morris et al., 2016). We pooled mean SRS *T* scores from Morris et al. (2016). and seven additional studies in NF1 cohorts (Chisholm et al., 2022; Huijbregts & de Sonneville, 2011; Lalancette et al., 2022; Lubbers et al., 2022; Morotti et al., 2021; Payne et al., 2020; van Eeghen et al., 2013) (*n* = 1046) and 2 studies in NS (Adviento et al., 2014; Naylor et al., 2023) (*n* = 96) and compared these data with those from Adviento et al. (2014). on the other RASopathies (*n* = 40 for CS and *n* = 49 for CFC), calculating 95%CI's assuming normal distributions. We could confirm that NF1 participants were least affected, with a mean total *T* score of 58.35 (95%CI 57.52;59.17). CS (61, 95%CI 57.90;64.10) and NS (65.34, 95%CI 61.89;68.79) had higher total *T* scores, and CFC (74, 95%CI 70.36;77.64) the highest. Thus, CFC mean total *T* score was significantly higher than those in all other RASopathies (two‐tailed *α* < 0.05). Furthermore, the NS total *T* score was significantly higher than the NF1 mean total *T* score. The result of the pooling of the SRS total *T* scores from different studies is shown in Figure 3. An overview of the SRS total *T* scores across syndrome groups and studies is provided in Figure S1 (Supplement S3).

#### 4.1.2 ASD classifications: Autism diagnostic observation schedule (ADOS), autism diagnostic interview‐revised (ADI‐R)

ADOS scores across studies are shown in Table 1. While data for each RASopathy are scarce, taken together the ADOS and ADI‐R scores and classifications reflect what would be expected based on the previously mentioned questionnaire data, that is CFC patients having higher ADOS scores and more often receiving an autism spectrum classification based on the ADOS and ADI‐R in comparison to other RASopathies. However, the ADOS scores of individuals receiving an ASD classification based on ADOS and ADI‐R were not significantly different between RASopathies.

**TABLE 1:** Mean ADOS scores across included studies.

Instrument	Score type	Group	Communication	Social	Total	Authors and year (*n* patients with ADOS data)
ADOS‐G	AS module 3	NF1	2.07^a^	4.19^a^		Garg, Green, et al. (2013) (*n* = 47)
	AS module 3	NF1+ASD	3.86	8.36		Garg, Green, et al. (2013) (*n* = 14)
	AS module 3	NF1+bASD	2.08	4.23		Garg, Green, et al. (2013) (*n* = 13)
	AS module 3	NF1‐ASD	0.8	1.25		Garg, Green, et al. (2013) (*n* = 20)

			Social affect	Restricted and repetitive behavior		
ADOS‐2	AS module 3	NF1	6.54^a^	1.10^a^	7.74^a^	Garg et al. (2016) (*n* = 96)^b^
	AS module 3	NS	4.84^a^	1.27^a^	6.11^a^	Garg et al. (2017) (*n* = 40)
	AS module 3	CFC	8.22	4.00	12.22	Garg et al. (2017) (*n* = 9)
	AS module 3	NF1 female	4.52	0.69	5.50	Garg et al. (2016) (*n* = 42)^b^
	AS module 3	NF1 male	8.11	1.41	9.48	Garg et al. (2016) (*n* = 54)^b^
	AS module 3	NF1+ASD	9.89/9.73	1.25/2.00	11.11/11.80	Garg et al. (2015) (*n* = 36) ^c^/Stivaros et al. (2018) (*n* = 30)
	AS module 3	NF1+ASD female	8.33	1.67	10.00	Garg et al. (2016) (*n* = 9)^b^
	AS module 3	NF1+ASD male	10.45	2.16	12.61	Garg et al. (2016) (*n* = 31)^b^
	AS module 2/3	NF1+ASD female	5.8	1.4	7.1	Chisholm et al. (2023) (*n* = 28)
	AS module 2/3	NF1+ASD male	8.9	1.2	10.1	Chisholm et al. (2023) (*n* = 34)
	AS module 3	NS+ASD	8.8	2.5	11.0	Garg et al. (2017) (*n* = 12)
	AS module 3	NS+bASD	4.0	0.8	4.7	Garg et al. (2017) (*n* = 12)
	AS module 3	NS‐ASD	2.5	0.7	3.0	Garg et al. (2017) (*n* = 16)
	AS module 3	iASD	7.29	2.05	9.34	Garg et al. (2015) (*n* = 186)^c^
	CSS	NF1	2.92	0.81	2.48	Lubbers et al. (2022) (*n* = 260)
	CSS	NF1+ASD	7.3/5.4/6.73	5.0/4.5/1.57	6.8/4.8/6.11	Geoffray et al. (2021) (*n* = 48)^d^/Chisholm et al. (2022) (*n* = 65)/Lubbers et al. (2022) (*n* = 52)
	CSS	NS+ASD	6.4	6.5	6.4	Geoffray et al. (2021) (*n* = 11)^d^
	CSS	CFC+ASD	5.3	8.3	6.4	Geoffray et al. (2021) (*n* = 7)^d^

*Note*: Available mean ADOS scores across the included studies. Sometimes the scores are stated in the article, and in other instances, they could be calculated from figures reported in the article. The ADOS‐G and ADOS‐2 have different subscales and scoring algorithms and are reported separately. Furthermore, each ADOS module has its own scoring algorithm and classification cut‐off. Based on an ADOS‐2 algorithm score and the individual's age a calibrated severity score can be calculated for comparison between different modules and ages. An ADOS‐2 module 3 algorithm total score ≥ 7 or a CSS ≥4 corresponds to an ASD classification.

Abbreviations: ADOS‐G, Autism Diagnostic Observation Schedule‐Generic; ADOS‐2, Autism Diagnostic Observation Schedule‐second edition; AS, algorithm score; CFC, cardio‐facio‐cutaneous syndrome; CFC+ASD, only CFC participants preselected on ASD; CSS, calibrated severity score; iASD, idiopathic ASD; NF1, neurofibromatosis type 1; NF1+ASD, only NF1 participants preselected on ASD; NF1+bASD, only NF1 participants preselected on broader ASD, excluding ASD itself; NF1‐ASD, only NF1 participants without ASD; NS, Noonan syndrome; NS+ASD, only NS participants preselected on ASD; NS+bASD, only NS participants preselected on broader ASD, excluding ASD itself.

^a^ Calculated from figures reported in the article. Because the figures in the article were rounded, the results may differ from those that would have been calculated based on the raw data.

^b^ Pooled data from Garg, Green, et al. (2013), and Stivaros et al. (2018).

^c^ Pooled data from Garg, Green, et al. (2013), Plasschaert et al. (2015), and Stivaros et al. (2018).

^d^ Pooled data from Garg, Green, et al. (2013), Garg et al. (2017), and Stivaros et al. (2018).

Garg et al. studied children with NF1 (*n* = 47) using both the ADI‐R and the ADOS (Garg, Green, et al., 2013). Using the Collaborative Program of Excellence in Autism (CPEA) criteria integrating both ADOS and ADI‐R (Lainhart et al., 2006), 29.8% of the participants had ASD, 27.7% broader ASD (bASD), and the remaining 42.5% were classified as non‐ASD. In a later study, Garg et al. used the same technique comparing CFC (*n* = 9) and NS (*n* = 40) (Garg et al., 2017). 88.9% of CFC patients met the criteria for ASD, and the remaining 11.1% was classified as bASD. In CS, 30% was classified as ASD and another 30% as bASD. As could be expected, CFC had higher total ADOS algorithm scores than NS (Lainhart et al., 2006). In a study of children with NF1 by Lubbers et al., 20.0% (52/260) received an ADOS autism spectrum classification (Lubbers et al., 2022).

Geoffray et al. analyzed ADOS and ADI‐R data from RASopathy participants with an ASD classification from previous studies (Garg et al., 2017; Garg, Green, et al., 2013; Geoffray et al., 2021; Stivaros et al., 2018). The ADOS total Calibrated Severity Score (a measure allowing comparisons between different modules) did not differ significantly between NF1+ASD (*n* = 48), NS+ASD (*n* = 11), and CFC+ASD (*n* = 7). Garg et al. compared ADOS data from previous studies (Garg, Green, et al., 2013; Plasschaert et al., 2015; Stivaros et al., 2018) of children with NF1+ASD (*n* = 36) to normative data of autism and autism spectrum from the ADOS manual (Garg et al., 2015; Lord et al., 2012). The total algorithm score in NF1+ASD was significantly lower than autism norms but did not differ significantly from autism spectrum norms.

Importantly, while the findings from ADOS and ADI‐R parallel those from the SRS and other questionnaires, their classifications do not completely capture the same entity. In the Eijk et al. study, for the majority of the participants (*n* = 103) SRS and ADOS scores were available and ADOS and SRS classifications disagreed in 25.2% or 16.5% of individual cases, depending on SRS cut‐off (total *T* score ≥60 or ≥76, respectively), each instrument classifying a number of individuals as non‐spectrum while the other instrument classified them in the autism spectrum (Page et al., 2021). Data from the Supplementary materials of Lubbers et al., expanding on the data from Eijk et al., indicate that both SRS and ADOS data were available for 206 NF1 participants (Lubbers et al., 2022). Using SRS total *T* score ≥60 as a cut‐off, there was disagreement going both directions in 22.8% of the cases. Furthermore, Chisholm et al., reported that only 33.8% and 63.1% of their sample of NF1 participants having an SRS total *T* score ≥60 met the ADI‐R and ADOS‐2 algorithm cut‐offs, respectively (Chisholm et al., 2022).

#### 4.1.3 Relationship of measuring instruments with clinical expert diagnosis

In the study by Eijk et al., 10.9% (14/128) of participants received a clinical DSM‐IV ASD diagnosis after systematic assessment (Eijk et al., 2018). This figure was lower than the proportion of children classified as autism spectrum according to the ADOS or the SRS. While negative predictive values were good (all >0.90), ADOS and SRS had a poor positive predictive value of clinical diagnosis (0.45 and 0.35/0.63 depending on SRS cut‐off, respectively) and this increased to only 0.71/0.75 when combining both. In the study by Alfieri et al., DSM‐IV‐TR criteria for ASD were met in 71% of CFC patients reaching the SCQ/M‐CHAT cut‐off (5 out of 7), while this was only the case in 25% of CS patients screening positive (1 out of 4), and none of the NS patients screening positive (0 out of 4) (Alfieri et al., 2014).

### 4.2 Detailed analysis of ASD profiles

#### 4.2.1 SRS

The SRS not only yields a total score, but also scores on five subscales: Social awareness, Social motivation, Social communication, Social cognition, and Autistic mannerisms. Comparison across these subscales indicates a similar profile in RASopathies compared to iASD, and in NF1 compared to other developmental pathology.

Taking the individuals with CFC, CS, NS, and NF1 scoring above the SCQ cut‐off together (*n* = 54) and comparing them to iASD (*n* = 167), Adviento et al. found the five SRS subscales to be affected in a similar pattern, with highest *T* scores on Autistic mannerisms, and lowest on Social awareness and Social motivation. Visual inspection of a diagram of the first two components of principal component analysis of SRS subscale scores showed that RASopathy+ASD and iASD did not cluster separately (Adviento et al., 2014). Research in minors with NF1 that were not selected for ASD (*n* = 52) showed the same pattern, with most impairment on Autistic mannerisms and least on social cognition and social motivation (Walsh et al., 2013). In contrast, a study by Plasschaert et al. found that children and adolescents with NF1 (*n* = 82) were most impaired on social cognition, social communication, and autistic mannerisms (Plasschaert et al., 2015). Van Eeghen et al. also reported most impairment in NF1 (*n* = 50) on Autistic mannerisms and Social cognition and least on Social awareness and they observed a similar profile in tuberous sclerosis complex (*n* = 64), non‐familial childhood‐onset epilepsy of unknown cause (*n* = 66), and iASD (*n* = 210) (van Eeghen et al., 2013). Garg et al. pooled data on NF1+ASD from several studies (*n* = 36) (Garg, Green, et al., 2013; Plasschaert et al., 2015; Stivaros et al., 2018), and found a similar pattern as the one reported by Plasschaert et al., with 67% exhibiting severe impairments on Autistic mannerisms, 53% on social cognition, 47% on Social communication, 44% on Social motivation and 42% on Social awareness (Garg et al., 2015).

#### 4.2.2 ADOS and ADI‐R

When the subdomains of the ADOS and the ADI‐R are considered there is again evidence of CFC patients having more ASD symptoms than other RASopathies. However, when only RASopathy patients with an ASD classification are being compared, differences between the RASopathies are not significant. Furthermore, based on one study comparing individual ADOS items NF1+ASD patients could have better eye contact and less stereotyped and repetitive language than iASD, but evidence from other studies to corroborate this finding is lacking. Only in NF1 a more detailed analysis of the profile of separate ADOS and ADI‐R items endorsed has been reported.

Garg et al. found that participants with CFC (*n* = 9) had higher ADOS Social affect (SA) and Restricted and repetitive behavior (RRB) subscale scores than NS (*n* = 40). The same pattern of higher scores in CFC was seen for all three domains of the ADI‐R (Garg et al., 2017). In analyses by Geoffray et al. individuals with NF1+ASD (*n* = 48) had lower levels of ADOS RRB compared to NS+ASD (*n* = 11) and CFC+ASD (*n* = 7), but this difference did not survive Bonferroni correction for multiple testing and on the ADI‐R RRB domain the NF1+ASD sample was most impaired, though this difference was not statistically significant either. ADOS SA and RRB in these RASopathies were comparable with data on iASD from two large cohorts (Geoffray et al., 2021; Hus et al., 2014; Hus Bal & Lord, 2015). Garg et al. found in another study that NF1+ASD (*n* = 36) had a significantly lower RRB algorithm score than autism spectrum norms from the ADOS manual, while total algorithm scores were not different. Further exploration by comparisons of all algorithm items separately found that on almost all the SA algorithm items the NF1+ASD group had significantly higher scores than the autism spectrum group, but significantly lower scores on Unusual eye contact. Comparisons of individual RRB algorithm items were not significant, except for the NF1+ASD group scoring lower on Stereotyped/idiosyncratic use of words and phrases. Compared to autism norms NF1+ASD had a significantly lower RRB algorithm score as well, and significantly lower scores on SA algorithm item Unusual eye contact and all RRB algorithm items (Garg et al., 2015). Lubbers et al. reported that their NF1+ASD group (*n* = 52) did not differ from iASD concerning ADOS Social affect and Restricted and repetitive behavior scores (Lubbers et al., 2022). Chisholm et al. found that in their NF1+ASD sample (*n* = 68) on the ADOS and the ADI‐R a number of algorithm items from both the A and the B domains were endorsed in more than 60%, including play with peers, reciprocal conversation, sharing, quality/appropriateness of social responses, difficulties with minor changes and sensitivity to noise. In contrast, some items were endorsed in <20% of cases, exclusively involving the B domain: mannerisms, unusual preoccupations, verbal rituals, unusual attachments, neologisms, idiosyncratic language, and immediate echolalia. Without comparison to other RASopathies and iASD this finding is not easy to interpret (Chisholm et al., 2022).

#### 4.2.3 Other instruments

Foy et al. compared NF1, NS, CS, CFC, and iASD using the Social Emotional Assets and Resilience Scales (SEARS) and found social competence and empathy in RASopathy patients to be better than in iASD (Foy et al., 2022). For both RASopathies and iASD, the ratings for empathy were higher than for social competence, but in al the RASopathies this difference was significantly larger than in iASD. In all RASopathies but CS, participants were more likely to have severe deficits in social competence than in empathy. The mean difference between the two SEARS sub‐scale scores was the largest in NS (6.1 points) followed by NF1 (5.9 points), CFC (5.3 points), and the smallest in CS (3.9 points). Those RASopathy participants who had a previous clinical diagnosis of ASD were as likely as the iASD group to have severe deficits in social competence, but were less likely to have severe deficits in empathy. Morotti et al. found significantly lower RRB in NF1 (*n* = 45) compared to ASD cases without NF1 (*n* = 180), according to the Repetitive Behavior Scale‐Revised (RBS‐R) (Morotti et al., 2021). Based on data from the Centers for Disease Control and Prevention Autism and Developmental Disabilities Monitoring Network Bilder et al. found that 8‐year‐old children with NF1+ASD (*n* = 22) significantly less often met DSM‐IV criterion 1a (Difficulty using or understanding non‐verbal communication) than their peers with only ASD (*n* = 12,249) (58% vs. 84%, respectively) (Bilder et al., 2016). Using the Sensory Profile (SP), NF1 participants were compared to healthy controls and were found to have more difficulties in sensory processing involving auditory, touch, movement, body position, and oral modalities (Pride et al., 2023). In comparison to published iASD data, they had similar scores on the quadrants Registration and Seeking, but lower on the quadrants Sensitivity and Avoidance. The SP quadrant scores were correlated with higher impairment in social responsivity (as measured with the SRS) and lower social skills (as measured with the SSIS), suggesting a connection between these different behavioral domains of ASD. There were no includable SP data on NS and CS, but Onesimo et al. reported a high degree (45%) of CFC participants (*n* = 27) having a score in the atypical range for the oral modality (Onesimo et al., 2023). Because this study focused on feeding skills, other modalities were not reported. Naylor et al. reported that, according to the KSADS, no NS participants met the DSM‐5 A criterion for ASD, but 9.5% met the B criterion (Naylor et al., 2023).

### 4.3 Moderating factors

An overview of the main outcomes regarding moderating factors is provided in Table 2.

**TABLE 2:** Main outcomes from studies investigating moderating factors.

Moderating factor	Main outcome
Age	In NF1 evidence for an age effect, but findings, including the direction of this effect, are conflicting. This could partly be attributed to the different instruments being used (Chisholm et al., 2023; Eijk et al., 2018; Glad et al., 2021; Haebich et al., 2022; Kenborg et al., 2021; Morris et al., 2016; Plasschaert et al., 2015; Pride et al., 2023; Tinker et al., 2014) In NS and CS possible age effect (ASD symptoms decreasing with age) attributable to small sample sizes and instrument properties (Niemczyk et al., 2015; Schwartz et al., 2017; Young et al., 2018)
Sex	ASD sex ratios in RASopathies are lower than in iASD Variable sex ratios in NF1 and NS, attributable to small sample sizes and different diagnostic criteria (Adviento et al., 2014; Eijk et al., 2018; Garg, Green et al., 2013; Garg et al., 2015; Garg et al., 2017; Geoffray et al., 2021; Hirabaru & Matsuo, 2018; Morris et al., 2016; Plasschaert et al., 2015; Stivaros et al., 2018) Sex ratio in CFC close to 1 and less variable, attributable to high fulfillment of ASD diagnosis (Adviento et al., 2014; Garg et al., 2017; Geoffray et al., 2021) *NF1* pathogenic variants increase ASD trait expressivity in both males and females (Constantino et al., 2015, Morris et al., 2016; Payne et al., 2020)
ADHD symptoms	In NF1 high correlation ADHD traits and ASD traits, in part due to instruments not being entirely specific for ADHD or ASD (Constantino et al., 2015; Morris et al., 2016; Walsh et al., 2013) In NF1 high comorbidity ADHD and ASD (Cohen et al., 2022; Garg, Green et al., 2013; Hirabaru & Matsuo, 2018; Walsh et al., 2013)
Cognition	In NF1 and NS ASD traits mostly independent of total IQ (Eijk et al., 2018; Garg et al., 2017; Haebich et al., 2022; Pride et al., 2013; van Eeghen et al., 2013) In NF1 executive functioning and ASD traits negatively correlated, but moderating effect of ADHD (Haebich et al., 2022; Huijbregts & de Sonneville, 2011; Loitfelder et al., 2015; Plasschaert et al., 2016)
Genotype	Unimodal distribution and positive shift of ASD traits in NF1, CS, NS, and CFC suggest that all pathogenic variants related to RASopathies increase ASD traits (Adviento et al., 2014; Constantino et al., 2015; Morris et al., 2016; Payne et al., 2020) Intraclass correlations between NF1 first‐degree relatives suggest mutation specificity for the amount of ASD traits (Constantino et al., 2015; Morris et al., 2016) Higher ASD traits in NF1 with microdeletions (Kehrer‐Sawatzki et al., 2020)

Abbreviations: CFC, cardiofaciocutaneous syndrome; CS, Costello syndrome; NF1, neurofibromatosis type 1; NS, Noonan syndrome.

#### 4.3.1 Age

The available data indicate a possible age effect in NF1, CS, and NS on ASD symptomatology, but some findings, including contradictions, may be attributable to differences between instruments.

In NF1 Glad et al. compared SSIS data from “early childhood” (3–6 years) and “school age” (9–13 years) (Glad et al., 2021). Early childhood and school‐age social skills were not significantly different. However, within the data from early childhood, there was a positive correlation of social skills with age, and social skills at 3 years were significantly lower than social skills at 6 years. Only social skills at 5–6 years correlated with social skills at school age. Haebich et al. found no significant correlation between SSIS scores and age in their NF1 sample (Haebich et al., 2022). Morris et al. found a peak in SRS total *T* scores at 8 to 17 years compared to younger children and adults (*n* = 531) (Morris et al., 2016). Including only participants younger than 18 years, Plasschaert et al. found significantly less individuals under 8 years with SRS *T* scores in the severe or clinically relevant range and a significant correlation between age and total raw SRS scores (*n* = 82) (Plasschaert et al., 2015). Tinker et al. screened children with NF1 (*n* = 67) for ASD, using the M‐CHAT in those younger than 4 years and the CAST in those being older. No child screened positive using the M‐CHAT, but 12.5% of the children screened using the CAST did. It is unclear if this finding is attributable to a coincidence, an age effect, or different psychometric properties of these instruments (Tinker et al., 2014). Eijk et al. found a significant age effect on DSM‐IV ASD diagnosis in children with NF1 aged 2 to 10 years old (*n* = 128). The mean age of children with NF1+ASD was 6.36 years, significantly older than children with NF1 without ASD (5.13 years) (Eijk et al., 2018). Chisholm et al. found SRS, ADI‐R, and ADOS‐2 scores to be not correlated with age in a group of children with NF1 aged 3–16 years who screened positive on the SRS (total *T* score ≥60) (Chisholm et al., 2023). When they compared lifetime and current ratings on the ADI‐R at the group level there was a significant abatement of symptoms in all three domains, and they found an interesting interaction between sex and age. There was a significant decline in RRB in both sexes, but a significant decline in impairment on the sub‐scales social and communication was only found in males. Individual trajectories of impairment in social communication varied widely, with some participants improving, others staying fairly constant, and some others deteriorating. When comparing to the general population, Kenborg et al. found only a statistically increased hazard ratio for ASD‐related hospital contacts in NF1 patients in the age group between 0 and 7 years (Kenborg et al., 2021). Pride et al. found sensory sensitivity measured using the SP to be not associated with age in their NF1 sample (Pride et al., 2023).

Schwartz et al. screened CS patients aged 22 months to 18 years for ASD (*n* = 14). In the group younger than 48 months the M‐CHAT was used, and, depending on using the recommended or a more conservative cut‐off, 71% (five out of seven) or 29% (two out of seven) screened positive, respectively. In contrast, children older than 48 months were screened using the M‐CHAT, but 0% (none out of seven) screened positive (Schwartz et al., 2017). In a reaction to this, Young et al. supplemented the CS data from Adviento et al. with new data (*n* = 53) and analyzed these in function of age. They found no significant correlation between SRS scores and age and, contrasting Schwartz et al.'s findings, the ASD rate according to the SCQ was higher in children older than 4 years (Young et al., 2018).

Niemczyk et al. administered the Developmental Behavior Checklist (DBC)‐Parent in children with NS from 5 to 17 years and the DBC‐Adult in adults with NS between 18 and 48 years (*n* = 29). 35% of children and 10% of adults reached the clinical cut‐off (Niemczyk et al., 2015).

#### 4.3.2 Sex

Reported male‐to‐female ASD ratios in different RASopathies are shown in Table 3. Whenever possible, we corrected ratios for the male‐to‐female ratio of the sample in which individuals with ASD were identified, assuming equal ratios of males to females in each RASopathy. Most data are available for NF1, and indicate a male predominance to a variable extent, with 10 studies discovering more males than females with NF1 displaying ASD according to ADOS, ADI‐R, SRS, SCQ, or clinical diagnosis (Adviento et al., 2014; Eijk et al., 2018; Garg et al., 2015, 2016; Garg, Green, et al., 2013; Geoffray et al., 2021; Hirabaru & Matsuo, 2018; Morris et al., 2016; Plasschaert et al., 2015; Stivaros et al., 2018). Only from one study, we deduced a male‐to‐female ratio in which females were slightly more represented (Chisholm et al., 2022). Corrected male‐to‐female ASD ratios in NF1 range from 0.97:1 to 3.04:1. Of note, even the higher end of this estimate is lower than the reliable estimate of the sex ratio in iASD of 3.3:1 (Loomes et al., 2017). It is worth noting that ratios based on SRS total *T* score cut‐offs lead to lower ratios than those based on ADOS and/or ADI‐R classifications. Unexpectedly, Kenborg et al. found no significant difference in hazard ratios for ASD‐related hospital contacts between males and females with NF1 in their cohort study (Kenborg et al., 2021).

**TABLE 3:** ASD sex ratios in RASopathies across the included studies.

Diagnostic instrument	NF1	CS	NS	CFC	Authors and year (*n* participants identified with ASD)
ADOS, ADI‐R, CPEA criteria	1.93:1^a^				Garg, Green, et al. (2013) (*n* = 47)
SCQ cut‐off	NA^b^	2.57:1	1.28:1	1.52:1	Adviento et al. (2014) (*n* = 7/11/10/29)
Clinical DSM‐IV diagnosis	3.02:1				Plasschaert et al. (2015) (*n* = 27)
SRS and ADOS cut‐off	3.00:1^c^				Garg et al. (2015) (*n* = 36)^d^
SRS, ADOS and ADI‐R cut‐off	2.68:1				Garg et al. (2016) (*n* = 40)^e^
SRS total *T* score threshold (60/76)	1.25:1/1.6:1				Morris et al. (2016) (*n* = 208/70)^f^
ADOS, ADI‐R, CPEA criteria			1.80:1	1.00:1	Garg et al. (2017) (*n* = 24/8)
Clinical DSM‐IV diagnosis	3.04:1				Eijk et al. (2018) (*n* = 14)
SRS *T* score threshold (60/76)	1.40:1/1.50:1				Hirabaru and Matsuo (2018) (*n* = 50/29)
ADOS, ADI‐R CPEA criteria	4.00:1^c^				Stivaros et al. (2018) (*n* = 30)
ADOS and ADI‐R cut‐off	3.00:1^c^		2.70:1	1.07:1	Geoffray et al. (2021) (*n* = 48/11/7)^g^
SRS *T* score threshold (60)	0.97:1				Chisholm et al. (2022) (*n* = 77)

*Note*: All ratios are male‐to‐female ratios and, whenever possible, corrected for the male‐to‐female ratio of the sample in which participants with ASD were identified, under the assumption of an equal number of males and females within each RASopathy population.

Abbreviations: ADI‐R, Autism Diagnostic Interview‐Revised; ADOS, Autism Diagnostic Observation Schedule; CFC, cardio‐facio‐cutaneous syndrome; CPEA, Collaborative Program of Excellence in Autism; CS, Costello syndrome; DSM‐IV, Diagnostic and Statistical Manual of Mental Disorders, 4th edition; NA, not applicable; NF1, neurofibromatosis type 1; NS, Noonan syndrome; SCQ, Social Communication Questionnaire; SRS, Social Responsiveness Scale.

^a^ Subsample from Garg, Lehtonen, et al. (2013)^39^, proportional to the original distribution categories of SRS scores. When corrected for the original male‐to‐female ratio in the sample of Garg, Lehtonen, et al. (2013) instead of the ratio in the subsample, ASD sex ratio is estimated at 2.00:1.

^b^ Not possible to calculate a sex ratio because only NF1 males (27% of males) scored above the SCQ cut‐off.

^c^ Uncorrected for male‐to‐female ratio of the sample in which the ASD participants were identified, as this was not reported.

^d^ Pooled data from Garg, Green, et al. (2013)^1^, Plasschaert et al. (2015)^10^, and Stivaros et al. (2018)^5^.

^e^ Pooled data from Garg, Green, et al. (2013)^1^, and Stivaros et al. (2018)^5^.

^f^ Pooled original data with those from Garg, Lehtonen, et al. (2013)^39^, Walsh et al. (2013)^31^, Adviento et al. (2014)^25^, Constantino et al. (2015)^27^, and Plasschaert et al. (2015)^10^.

^g^ Pooled data from Garg, Green, et al. (2013)^1^, Garg et al. (2017)^3^, and Stivaros et al. (2018)^5^.

Not only do NF1 males more often receive an ASD diagnosis than females, most evidence also indicates more ASD symptoms in NF1 males. Garg et al. found higher scores in male than female NF1 (*n* = 194) on all total scores and subscales of SRS, ADOS and ADI‐R, which after Bonferroni correction for multiple testing was still significant for all SRS raw scores, ADI‐R subscales A1 (non‐verbal behaviors), A3 (shared enjoyment), A4 (socio‐emotional reciprocity), B2 (conversational interchange), B3 (stereotyped, repetitive or idiosyncratic speech), C1 (preoccupation or circumscribed pattern of interest), and the ADOS SA and total algorithm scores. Including only participants meeting the criteria for a research diagnosis of ASD, after Bonferroni correction males only had significantly higher scores than females on ADI‐R subscales A1 and B2 (Garg et al., 2016). Chisholm et al. studied children with NF1 who screened positive on the SRS (total *T* score ≥60) and found no significant differences between males and females in the ADI‐R and ADOS RRB domains (Chisholm et al., 2023). In contrast, males had higher impairment on the ADI‐R Social and Communication domains, and on the ADOS Social affect sub‐scale. This resulted in higher proportions of males exceeding ADI‐R Social and Communication cut‐offs and ADOS autism spectrum cut‐offs. Even though roughly twice as many males fulfilled all three ADI‐R sub‐scale cut‐offs, this difference was not statistically significant. Based on the difference between lifetime and current scores on the ADI‐R subscales there was a decline in RRB in both sexes, but on the sub‐scales Social and Communication only in males. In four other studies, SRS *T* scores in NF1 did not differ between males and females (Constantino et al., 2015; Morris & Gutmann, 2018; Payne et al., 2020; van Eeghen et al., 2013). Nevertheless, Morris et al. found significantly higher total *T* scores and subscale scores in males compared to females in their meta‐analysis (*n* = 531) (Morris et al., 2016). In another study, males had significantly more often *T* scores of 60 or higher on the SRS subscales Social motivation and Social communication (*n* = 66) (Walsh et al., 2013). Using the SPSS Pride et al. found that males with NF1 exhibited significantly less observer‐rated prosocial behavior than females (*n* = 62). This sex difference was absent in the control group (*n* = 39) (Pride et al., 2013). Three studies showed continuously, unimodally distributed SRS *T* scores in both males and females, suggesting a pathological shift in NF1 participants as a whole (Constantino et al., 2015; Morris et al., 2016; Payne et al., 2020). Glad et al. found no significant difference in social skills between male and female NF1 participants using the SSIS (Glad et al., 2021). Haebich et al., on the contrary, found a significant but weak correlation with sex (girls having higher social skills) in their NF1 sample (Haebich et al., 2022). According to the study by Pride et al. using the SP, sensory sensitivity in children with NF1 is not related to sex (Pride et al., 2023).

Considering other RASopathies, one study found a non‐significant difference in the proportion of males and females reaching the SCQ threshold (1.28:1) in NS (*n* = 52) (Adviento et al., 2014). However, estimates of the male‐to‐female ASD ratios in NS according to ADOS and ADI‐R were higher. While Garg et al. (*n* = 40) used the CPEA criteria and found a sex ratio of 1.80:1, Geoffray et al. (*n* = 11) required meeting both the ADI‐R and ADOS cut‐off and found a sex ratio of 2.70:1 (Garg et al., 2017; Geoffray et al., 2021). These three studies also included CFC participants and found no significantly elevated male bias (Adviento et al., 2014; Garg et al., 2017; Geoffray et al., 2021). In CS, one study (*n* = 44) found a non‐significantly elevated male‐to‐female ratio using the SCQ of 2.57:1 (Adviento et al., 2014).

#### 4.3.3 Attention‐deficit/hyperactivity disorder (ADHD)

Findings on the relationship between ADHD and ASD in NF1 are shown in Table 4. Based on these findings, a high comorbidity between ASD and ADHD is evident, with a correlation between ASD and ADHD symptoms. Cohen et al. reported that the 10% of their NF1 sample who met the SCQ cut‐off for ASD already had a clinical ADHD diagnosis (Cohen et al., 2022). Indicating high comorbidity, Garg et al. found 40.3% of their NF1 sample (*n* = 109) to have both ASD and ADHD according to the SRS (total *T* score >60) and the Conners' Parent Rating Scale‐Revised, and 18.2% to have ASD without ADHD. Using a more conservative SRS *T* score cut‐off of 76, these figures became 25% and 4.8%, respectively (Garg, Lehtonen, et al., 2013). Hirabaru and Matsuo (*n* = 143) found the same pattern using the SRS cut‐off of 76: 18.9% with ASD and ADHD, and only 1.8% with ASD without ADHD (Hirabaru & Matsuo, 2018). *T* scores higher than 60 on the SRS Total scale and the Social cognition and Social communication subscales were found significantly more frequently in NF1 patients with a research diagnosis of ADHD compared to NF1 patients without ADHD by Walsh et al. (*n* = 66). After correction for false discovery rate, significant correlations were found between the Inattention score of the Vanderbilt ADHD Diagnostic Parent Rating Scale (VADPRS) and all SRS subscales, except Social awareness. There was a significant correlation between the Hyperactivity/impulsivity VADPRS score and the SRS Social cognition subscale as well (Walsh et al., 2013). Three studies found positive correlations in NF1 between SRS total *T* scores and different versions of the Conners ADHD rating scales, but the two variables displayed different distributions. SRS scores were unimodally distributed, whereas ADHD indices were bimodal, implying comorbidity instead of confounding (Constantino et al., 2015; Morris et al., 2016; Payne et al., 2020). Another study (*n* = 122) found a moderate positive correlation between both Conners' parent scales inattention and hyperactivity/impulsivity *T* scores, and SRS *T* scores. SSIS scores were also moderately correlated with inattention, and weakly with hyperactivity/impulsivity (Payne et al., 2020). Using the same instruments Haebich et al. found moderate to strong intercorrelations between ADHD symptoms and social skills (Haebich et al., 2022). In their analyses, ADHD symptoms mediated the relationship between executive functions and social skills. Glad et al. found that SSIS social skills in early childhood were negatively correlated to Conners' hyperactivity and inattention at the same age (Glad et al., 2021). At school age, social skills were negatively correlated with both inattention in early childhood and inattention and hyperactivity at school age. Morotti et al. applied the SRS in children with NF1 (*n* = 45) and typically developing children (*n* = 180). They found 30% of the significant difference in SRS total *T* score to be explained by CBCL ADHD subscale scores, and after analysis of covariance with the CBCL ADHD subscale, CBCL Internalizing scale, and Vineland Communication standard score, the difference became negligible. Stepwise multiple logistic regression with high or low SRS *T* score as dependent variable retained CBCL ADHD and Internalizing and Vineland Communication standard scores as significant predictors, but not group status (Morotti et al., 2021). When classifying based on the ADI‐R and ADOS‐G, Garg et al. (*n* = 47) found no significant differences in parent‐ and teacher‐rated ADHD symptoms between children with NF1+ASD, NF1+bASD and NF1 without ASD (Garg, Green, et al., 2013). Chisholm et al. found a weak correlation between hyperactivity/impulsivity and ADI‐R scores, but no correlation between ADHD symptoms and ADOS‐2 scores (Chisholm et al., 2022). Concerning sensory processing, Pride et al. found SP quadrant scores to be positively correlated with both inattention and hyperactivity/impulsivity (Pride et al., 2023).

**TABLE 4:** Effect ADHD on ASD outcome in NF1.

ADHD instrument	ASD instrument	Reported relationship between ADHD and ASD	Authors and year
CPRS‐R, CTRS‐R	ADI‐R, ADOS‐G	Equal ADHD symptoms in NF1+ASD, NF1+bASd, and NF1 without ASD	Garg, Green, et al. (2013)
CPRS‐R	SRS	Using SRS cut‐off ≥60: 40.3% ASD+ADHD and 18.2% ASD without ADHD Using SRS cut‐off ≥76: 25% ASD+ADHD and 4.8% ASD without ADHD	Garg, Lehtonen, et al. (2013)
VADPRS/taking stimulant medication	SRS	In NF1+ADHD significantly more often total *T* score, social cognition, and social communication >60 than in NF1‐ASD	Walsh et al. (2013)
Conners‐3, CAARS	SRS	Moderate positive correlation between SRS total *T* score and ADHD measures, but different distribution	Constantino et al. (2015)
CAARS, CADS, Conners‐3, CPRS	SRS	Moderate positive correlation between SRS total *T* score and ADHD measures, but different distribution and sex ratio	Morris et al. (2016)^a^
ADHD‐RS4	SRS	Using SRS cut‐off ≥76: 18.9% ASD+ADHD and 1.8% ASD without ADHD	Hirabaru and Matsuo (2018)
CADS, Conners‐3	SRS, SSIS	Moderate positive correlation between SRS total *T* score and both Inattention and Hyperactivity/impulsivity	Payne et al. (2020)
CBCL	SRS	Significant positive correlation between SRS and CBCL ADHD subscale 30% of SRS difference between NF1 and TD accounted for by CBCL ADHD In the regression model CBCL ADHD, CBCL Internalizing, and Vineland Communication significant predictors of SRS score, but not NF1/TD group status	Morotti et al. (2021)
CPRS, Conners‐3	SSRS, SSIS	Hyperactivity and inattention in early childhood negatively correlated with social skills in early childhood Both inattention in early childhood and hyperactivity and inattention at school age negatively correlated with social skills at school age	Glad et al. (2021)
CADS, Conners‐3	SRS, ADOS‐2, ADI‐R	Both hyperactivity/impulsivity and inattention weak to moderately positively correlated with SRS total *T* scores Hyperactivity/impulsivity weakly positively correlated with ADI‐R scores No correlations between ADHD symptoms and ADOS‐2 scores	Chisholm et al. (2022)
VABS‐II, DSM‐IV‐TR	SCQ	All participants screening positive on the SCQ had a diagnosis of ADHD	Cohen et al. (2022)
SDQ	SEARS‐P	Higher hyperactivity/inattention predictive of both lower social competence and lower empathy	Foy et al. (2022)
CADS, Conners‐3	SSIS	Moderate to strong intercorrelations between social skills, ADHD symptoms, and executive functions ADHD symptoms mediate the relationship between executive functions and social skills	Haebich et al. (2022)

Abbreviations: ADHD‐RS4, ADHD Rating Scale 4; ADI‐R, Autism Diagnostic Interview‐Revised; ADOS‐G, Autism Diagnostic Observation Schedule‐Generic; CAARS, Conners Adult ADHD Rating Scale; CADS, Conners ADHD/DSM‐IV Scales; CBCL, Child Behavior Checklist; Conners‐3, Conners parent report form; third edition; CPRS‐R, Conners Parent Rating Scale–Revised; CTRS‐R, Conners Teacher Rating Scale‐Revised; NF1+ASD, only NF1 participants preselected on ASD; NF1+bASD, only NF1 participants preselected on broader ASD, excluding ASD itself; NF1‐ASD, only NF1 participants without ASD; SDQ, Strengths and Difficulties Questionnaire; SRS, Social Responsiveness Scale; TD, Typically developing; VABS‐II, Vineland Adaptive Behavior Scales‐2nd edition; VADPRS, Vanderbilt ADHD Diagnostic Parent Rating Scale.

^a^ Pooled original data with those from Garg, Lehtonen, et al. (2013)^39^, Walsh et al. (2013)^31^, Adviento et al. (2014)^25^, Constantino et al. (2015)^27^, and Plasschaert et al. (2015)^10^.

One study comparing ADHD rate in NS (*n* = 40) with and without ASD found no difference (Garg et al., 2017).

#### 4.3.4 Cognition

Total Intelligence Quotient was not found to be a significant modifier of ASD symptomatology in almost all studies involving NF1 and NS (Eijk et al., 2018; Garg et al., 2017; Pride et al., 2013; van Eeghen et al., 2013), but was not investigated in CS and CFC. Only Haebich et al. found a significant but weak positive correlation between SSIS social skills and full‐scale IQ in NF1 (Haebich et al., 2022). Furthermore, Pride et al. found a weak negative relationship between full‐scale IQ and SP quadrants Sensory registration and Sensory seeking (Pride et al., 2023). According to Garg et al., verbal IQ and SRS *T* scores were significantly correlated in NF1 (*n* = 36) (Garg et al., 2015).

Four studies looked at the association between executive functioning and ASD symptoms in NF1. The first study measured the correlation between SRS scores and five tests from the Amsterdam Neuropsychological Tasks (*n* = 30). The researchers found significant correlations between ASD traits and information processing speed, social information processing, cognitive control, and total cognition. Autistic traits remained significantly higher in NF1 compared to HC after correction for total cognition (Huijbregts & de Sonneville, 2011). Because of these correlations, a second study (*n* = 14) corrected SRS scores for the total score on the parent‐rated Behavior Rating Inventory of Executive Function (BRIEF) and the cognition scale of the Dysexecutive Questionnaire, but the SRS Total score and subscales Social motivation and Autistic mannerisms remained significantly higher than in HC (Loitfelder et al., 2015). The third study (*n* = 42) compared executive functioning in NF1 with iASD and HC using an extensive test battery involving inhibition, flexibility, generativity, spatial working memory, and planning, and the BRIEF (Plasschaert et al., 2016). Because many differences in EF remained after correction for IQ and SRS scores, executive functioning was considered a core feature in NF1 and ASD symptomatology was not corrected for executive functioning. Finally, as already mentioned in the section on ADHD symptoms, Haebich et al. found significant moderate to strong intercorrelations between social skills measured with the SSIS, ADHD symptoms according to the Conners rating scales, and executive functions measured with the BRIEF. According to mediation analyses, there was an important direct effect of executive functions on social skills, but also a mediating effect of both inattention and hyperactivity/impulsivity (Haebich et al., 2022).

#### 4.3.5 Genotype

Multiple authors reported an unimodal distribution of SRS *T* scores in NF1 in combination with significantly higher mean scores than population norms (Constantino et al., 2015; Morris et al., 2016; Payne et al., 2020), implying that all pathogenic *NF1* variants are associated with an increase in ASD traits. One study reported the same phenomenon in NS, CFC, and CS (Adviento et al., 2014). The lowest variance was seen in the group with CS, which is also the RASopathy with the least heterogeneous pathogenic variants of these three syndromes (Grant et al., 2018). Intraclass correlations between first‐degree relatives with NF1 found high correlations, suggesting specificity of *NF1* pathogenic variants for the increase in ASD symptoms (Constantino et al., 2015; Morris et al., 2016). One study found NF1 patients with a microdeletion (*n* = 30) to have a higher level of ASD symptoms compared to NF1 patients with an intragenic variant according to the SRS (Kehrer‐Sawatzki et al., 2020). One study (*n* = 57) detected significantly lower SRS *T* scores in NF1 caused by a variant in the 5′‐end of the *NF1* gene, with scores in the normal range compared to participants with a variant in the 3′ end (Morris & Gutmann, 2018). In the study by Alfieri et al., comparison of SCQ/M‐CHAT scores between NS (*n* = 38) caused by a variant in the *PTPN11, SOS1*, and *RAF1* genes showed no significant difference (Alfieri et al., 2014).

## 5 DISCUSSION

### 5.1 ASD profiles in RASopathies

With this systematic review, we investigated if RASopathies have a distinct ASD profile compared to iASD and whether a specific RASopathy is associated with its own ASD profile. RASopathy patients on average display more ASD features than US and the general population, and less than iASD. For all RASopathies, there is evidence for more individuals fulfilling an ASD diagnosis than in the general population. The available evidence does not support the existence of an ASD profile in RASopathies that differs from the one observed in iASD. However, because the current literature contains many limitations and inconsistencies, a distinct ASD profile in RASopathies cannot be ruled out.

Based on studies that directly compare RASopathies CFC patients have the highest levels of ASD features and most frequently receive an ASD classification, while NF1 patients have the lowest levels and are least likely to receive an ASD classification. Findings for NS and CS are situated in between. While quantitative differences in ASD traits between the RASopathies exist, these differences become non‐significant when only individuals with both a RASopathy and ASD are compared. Here too, the available literature does not allow us to confirm that RASopathies differ in terms of their ASD profile, without being able to rule out this possibility. Once again, the lack of a clear conclusion can largely be traced back to a number of inherent limitations in the research literature. A number of these limitations are discussed in a separate section below.

### 5.2 Moderating factors

#### 5.2.1 Age

There exists evidence for an age effect on ASD traits in NF1, but the direction of this effect depends on the instrument being used. While SRS data suggest that the amount of ASD symptoms is the highest between 8 and 17 years (Morris et al., 2016; Plasschaert et al., 2015), ADI‐R data suggest an improvement in impairment on the ASD symptom domains after 4–5 years of age (Chisholm et al., 2023). Furthermore, parents report most impairment in social skills on the SSIS at 3 years of age (Glad et al., 2021). Both the SRS and the SSIS have recently been recommended to measure social functioning in NF1 (Janusz et al., 2021), but while the SRS was specifically designed to capture impairments in social interaction and communication related to ASD, the SSIS is a measure of broader social functioning. This different scope may in part explain the contradictory findings, but an increase in ASD‐related behavior as picked up by the SRS remains difficult to reconcile with an improvement in social functioning measured by the SSIS. Other studies used different instruments depending on age, making differences between age groups hard to interpret (Tinker et al., 2014). Clearly, further research evaluating ASD symptoms across a wide age range with an instrument that performs reliably at all ages is most welcome. Longitudinal data instead of cross‐sectional measurements would be ideal to assess age effects on ASD symptoms in NF1. Findings in other RASopathies are limited and inconclusive, mainly because possible age effects can also be explained by differences between instruments and small sample sizes (Niemczyk et al., 2015; Schwartz et al., 2017; Young et al., 2018).

#### 5.2.2 Sex

In NF1 more males than females are classified as having ASD, but the reported male‐to‐female ASD ratio is variable and smaller than in iASD (Adviento et al., 2014; Eijk et al., 2018; Garg et al., 2015, 2016; Garg, Green, et al., 2013; Geoffray et al., 2021; Hirabaru & Matsuo, 2018; Morris et al., 2016; Plasschaert et al., 2015; Stivaros et al., 2018). Moreover, NF1 studies found higher total scores and sub‐scores on the SSIS, SRS, ADOS, and ADI‐R in boys compared to girls. Most of these differences disappear when only participants with a research diagnosis of ASD are compared (Chisholm et al., 2023; Garg et al., 2016; Morris et al., 2016; Pride et al., 2013; Walsh et al., 2013). *NF1* pathogenic variants increase the expressivity of ASD traits in both males and females (Constantino et al., 2015; Morris et al., 2016; Payne et al., 2020). In NS, all included studies find a male predominance, but the sex ratios are variable (Adviento et al., 2014; Garg et al., 2017; Geoffray et al., 2021). Estimates of the ASD sex ratio in CFC are all close to one (Adviento et al., 2014; Garg et al., 2017; Geoffray et al., 2021). The variability in ASD sex ratios in NF1 and NS can at least partly be explained by differences in methodology to attribute ASD status. An important issue in studying sex differences in ASD traits is the choice to use norm scores that have been adjusted for sex or not. Some norm scores (e.g., SRS *T* scores) take the generally better social and communicative functioning of females into account. This method increases the sensitivity to detect an increase in ASD traits in females but in doing so tends to diminish differences between males and females. The ADI‐R and ADOS algorithms, in contrast, have no separate cut‐offs for males and females. Of course, this does not completely rule out the possibility that parents and clinicians rate the same behaviors differently depending on sex. However, this difference could explain why estimates of ASD sex ratios in NF1 based on SRS cut‐offs are closer to one than those based on ADOS and/or ADI‐R. Chisholm et al. also note that in their study comparing boys and girls with NF1 selected on exceeding the SRS *T* score cut‐off boys still have higher ADI‐R and ADOS scores, which may in part be due to the fact that the SRS‐selected boys with a slightly higher level of ASD traits than that in the girls (Chisholm et al., 2023). The appropriateness of the choice to use norms based on biological sex is related to the precise question to which an answer is sought: comparing symptomatology between men and women within the same population, detecting a difference between two groups of mixed gender, screening for possible ASD in an individual, or something else.

In the case of CFC however, due to the generally high symptom burden, the majority of patients received an ASD diagnosis regardless of study criteria, explaining why the sex ratio is closer to one and less variable.

#### 5.2.3 Cognition

ASD traits in NF1 and NS were mostly found to be independent of total IQ, and whenever significant correlations were found, these were weak (Eijk et al., 2018; Garg et al., 2017; Pride et al., 2013; van Eeghen et al., 2013). However, the majority of NF1 patients have a total IQ in the normal range (TIQ > 70), and it is possible that the impact of total IQ is more pronounced at extreme values. An indication for this is NF1 patients with an *NF1* microdeletion having more often a TIQ < 70 in combination with higher SRS scores in comparison to NF1 patients with other pathogenic variants (Kehrer‐Sawatzki et al., 2020). In NF1 ASD traits and aspects of executive functioning are negatively correlated, but deficits in executive functioning do not completely explain the high ASD symptomatology. The relationship between executive functioning and ASD symptoms is mediated by ADHD symptoms (Haebich et al., 2022; Huijbregts & de Sonneville, 2011; Loitfelder et al., 2015; Plasschaert et al., 2016). Our review did not include studies on the correlations between intelligence, adaptive or executive functioning, and ASD traits in CS or CFC. Because total IQ and adaptive functioning are generally lower in these syndromes (Axelrad et al., 2011; Pierpont et al., 2014) and instruments such as the SRS tend to yield higher scores in the case of intellectual disability or impairment in adaptive functioning (Havdahl et al., 2016; Hus et al., 2013), significant correlations could be expected. The higher burden of ASD traits in CFC in comparison to other RASopathies could be attributable to lower IQ, but we found no data to corroborate this.

#### 5.2.4 ADHD

Evidence in NF1 points toward a correlation between measures of ADHD and ASD traits (Constantino et al., 2015; Morris et al., 2016; Walsh et al., 2013). However, instruments are not entirely specific for ASD or ADHD this is to be expected (Grzadzinski et al., 2016; Havdahl et al., 2016; Hus et al., 2013) and the literature also indicates a high comorbidity in NF1 (Cohen et al., 2022; Garg, Lehtonen, et al., 2013; Hirabaru & Matsuo, 2018; Walsh et al., 2013). There is an intercorrelation between measurements of ASD traits, ADHD symptoms, and executive functioning in NF1 (Haebich et al., 2022). While these concepts are theoretically distinct, it is a challenge to unambiguously attribute observable behaviors to only one of them, even leading to some authors questioning if there is a “true” increase in ASD symptoms in NF1 or if elevated scores can be attributed to confounding with ADHD (Fombonne et al., 2021; Morotti et al., 2021). Furthermore, it can be argued that statistically correcting one variable for correlations with another artificially reduces the clinical phenotype of NF1 consisting of impairments on multiple domains (Morris et al., 2021).

#### 5.2.5 Genotype

The unimodal, pathologically shifted distributions of ASD features in NF1, NS, CS, and CFC suggest that all pathogenic variants involved lead to an increase in ASD traits. However, in one study higher quantitative autism traits were detected in NF1 subjects with a variant at the 3′ end, as opposed to variants at the 5′ end of the *NF1* gene (Morris & Gutmann, 2018). In addition, intraclass correlation between NF1 first‐degree relatives suggested mutation specificity for quantitative ASD traits (Constantino et al., 2015; Morris et al., 2016). *NF1* microdeletions lead to a higher burden of quantitative autism traits, which is in line with the more severe somatic phenotype and a higher prevalence of neurodevelopmental disorders in these patients, reported in the same study (Kehrer‐Sawatzki et al., 2020). Because these patients with a microdeletion also have a lower IQ, it is unclear if the elevated SRS scores mainly reflect an effect of cognition, higher ASD symptom burden, or a combination of both.

### 5.3 Limitations of the included literature

The number of participants with NS, CFC, and CS in the included studies is minor compared to NF1 participants, even though NS (prevalence 1:1000–1:2500) is estimated to be more frequent than NF1 (prevalence 1:2600–1:3000) (Jafry & Sidbury, 2020). This makes comparisons between NF1 and other RASopathies challenging and makes it tempting to generalize findings from NF1 to other RASopathies. An important amount of research results has already been published describing the ASD phenotype of NF1, while this cannot be said about the other RASopathies. Another striking finding is that children and young people are better represented in published research than adults, which poses the risk of extending conclusions based on minors to the adult population with RASopathies. Although we decided to include studies published from 1994 onwards, we note that the oldest publication we could include in our review dates from 2011. In our opinion, this illustrates that only quite recently has a clear interest in ASD in RASopathies, coupled with a systematic research effort, emerged. However, a review by Chisholm et al. does suggest that active research was already being conducted into broader social functioning within NF1 in the preceding period (Chisholm et al., 2018).

While we already reduced the amount of research instruments through our exclusion criteria, different instruments measuring ASD symptoms are used across studies, each highlighting different behavioral aspects and complicating comparisons. Furthermore, most data consist of a general measurement of ASD symptoms and do not allow a fine‐grained appreciation of different ASD sub‐domains. When more refined analyses were performed than just determining the total quantity of ASD symptoms, this was often at the level of the A and B domains of the DSM‐5 criteria. At the same time, it is already clear based on these criteria themselves that the A and B domains are composed of very different behaviors that can be further broken down. The DSM‐5 criteria for ASD underwent an important change compared to the DSM‐IV in that abnormalities in sensory sensitivity were also included. Yet the only 2 studies that specifically investigated this domain in RASopathies and that we were able to include only dated from 2023 (Onesimo et al., 2023; Pride et al., 2023). The included studies were flawed due to small sample sizes, inconsistent investigation of modifiers, and inconsistent use of HC and iASD controls, compromising our analysis and reducing the precision of effect estimates. In some studies, bias arose due to retrospective review of medical documents, parent‐reported previous diagnosis of ASD, and in‐depth analysis of ASD features exclusively in participants who screened positive on screening instruments. A major limitation of studies comparing RASopathies+ASD is that they often analyze scores on the instrument used to assign the ASD classification (e.g., scoring above the cut‐off on the SRS or the ADOS/ADI‐R). Against the background of a rather continuous quantitative distribution of ASD symptoms in the population, the precise reason for doing this is often poorly substantiated but it also reduces an important part of the variation and lessens the chance of finding a statistically significant difference. Some studies did not find significant differences comparing SRS *T* scores between males and females (Constantino et al., 2015; Morris & Gutmann, 2018; Payne et al., 2020; van Eeghen et al., 2013). Because norms to convert raw scores into *T* scores differ by sex, this could have reduced existing differences.

Essential differences exist between studies in how participants were “diagnosed” with ASD. Sometimes this was a research classification based on either screening instruments, in‐depth assessment, or a combination of both. In other cases, this was a clinical diagnosis. Caution should be taken in the interpretation of results of screening instruments since specificity is low and ASD criteria are not elaborated. It can be questioned to what extent different measuring instruments and clinical assessments capture the same entity. This issue was illustrated by the few studies that reported the relationship between SCQ/M‐CHAT, SRS, and ADOS, on the one hand, and clinical diagnosis on the other hand (Alfieri et al., 2014; Eijk et al., 2018). In a recent study, an interesting fine‐grained analysis of endorsement of ADI‐R and ADOS items was done in a group of NF1 participants selected based on an SRS total *T* score ≥ 60 (Chisholm et al., 2022). The authors acknowledged this makes their sample not representative of all children with NF1, but were confident that they selected the majority of children that would show significant features of ASD on the ADI‐R or the ADOS. Given the fact that SRS and ADOS have been shown to disagree on classifications in both directions (Eijk et al., 2018; Lubbers et al., 2022), this assumption is debatable.

Incomplete molecular confirmation may have resulted in the inclusion of participants with an incorrect clinical RASopathy diagnosis and constrained the possibility to investigate genotype–phenotype correlations. The lack of molecular confirmation was most remarkable in NF1 participants, as the *NF1* gene was already cloned in 1990 (Viskochil et al., 1990). Diagnostic criteria for NF1 were until recently based on clinical findings and did not mention genetic status (National Institutes of Health, 1988). In the revised criteria, a pathogenic *NF1* variant has been adopted, but it is still not a necessary criterion (Legius et al., 2021). Furthermore, as the authors of the revised criteria admit, patients with the much rarer Legius syndrome, caused by a pathogenic variant in the *SPRED1* gene, meet these criteria for NF1 as well. One to 2% of patients with a clinical diagnosis of NF1 are estimated to carry pathogenic variants in *SPRED1* (Muram‐Zborovski et al., 2010). No study included in this review makes a reference to the new diagnostic criteria for the inclusion of participants, and it is not clear if they will have an influence on the degree of molecular confirmation of NF1 diagnosis in future research.

### 5.4 Implications for clinical practice and future research

Because of the high incidence of ASD symptoms in RASopathies clinicians should screen with low thresholds. However, they should be careful when interpreting screening results because in‐depth assessment of individuals who screen positive shows that many do not meet the diagnostic criteria. Screening instruments are too little specific and capture developmental problems in RASopathies that are not related to an ASD diagnosis. Data to widely implement genotype–phenotype correlation concerning ASD in RASopathies into clinical practice and counseling is currently lacking.

Future studies should compare sufficiently sized samples of different molecularly confirmed RASopathies, describing the observed ASD phenotype more extensively. This involves in‐depth assessment of all participants. A broader arsenal of instruments should be used, with full reporting of all subscales to comprehensively cover the different diagnostic criteria of ASD. To inform clinical practice, comparing the data from these instruments with systematic clinical assessments of these patients would be particularly relevant. However, when trying to understand the relationship between RASopathies and ASD symptoms, considering the indications for a continuous, unimodal distribution of ASD symptoms in these syndromes, the unclear relationships between instrument classifications and clinical diagnoses, and the statistical limitations caused by delineating subgroups for analysis, it seems advisable to take a more dimensional approach, studying the entire RASopathy population, regardless of ASD status. Researchers should appreciate the variability observed between and within syndromes. This heterogeneity invites us to not only look at the mutation in the Ras/MAP‐kinase pathway but also to consider other factors that shape the phenotype: genetic and environmental factors, level of cognitive impairment, and adaptive functioning. Consistent analysis and reporting of influence of gender, age, executive functioning, and ADHD should be considered. Prospective or repeated cross‐sectional study designs are warranted to map age effects. The study sample and the choice of the comparison group should also be tailored to the specific research question at hand. When we want to investigate the effect of RAS/MAPkinase pathway mutations on ASD symptoms irrespective of ASD diagnosis and distinguish this from the effect of other genetic and environmental influences, in most cases unaffected siblings are the preferred comparison group, but a comparison group from the general population that is matched on important parameters such as gender and age can also serve. If we specifically want to know whether the ASD symptom profile of persons with RASopathy and ASD differs from that in iASD, an iASD comparison group is logical, but it would be particularly useful if the diagnosis of ASD in both groups was made in the same way, for example after clinical assessment. In the included literature, RASopathy patients are often selected for ASD on the basis of a measuring instrument, while in the iASD group, the diagnosis was often made after a clinical assessment. These recommendations are summarized in Table 5.

**TABLE 5:** Recommendations for future research on ASD in RASopathies.

Molecular confirmation of RASopathy diagnosis Sufficiently sized samples for the effect being studied Systematic in‐depth assessment of all participants, regardless of screening status Use of a broad arsenal of validated instruments to comprehensively cover the different behavioral aspects of ASD Dimensional approach of ASD symptoms Contrasting of research findings with clinical assessment of participants Systematic measurement and reporting of possible modifiers (e.g., age, sex, cognition, ADHD symptoms) Prospective or repetitive cross‐sectional designs to assess age effects Matching the study sample and comparison group to the main research question

An interesting objective for future research that even goes beyond a better understanding of ASD in RASopathies is to develop treatments and interventions for individuals with RASopathies and ASD, determine how effective these are, and how their effectiveness can be improved. In this context, we should not lose sight of the fact that improving overall functioning and quality of life is probably an even more relevant endpoint than reducing ASD symptoms.

## 6 CONCLUSION

This systematic review indicates an elevated level of ASD traits in RASopathies compared to the general population. RASopathies differ from each other regarding the amount of ASD traits, while a qualitative difference in ASD symptoms is not supported by the data. Furthermore, as in the general population, ASD symptoms are unimodally and continuously distributed in RASopathy patients. The current literature does not provide sufficient evidence to indicate a specific ASD profile in RASopathies, but also contains many gaps and methodological limitations. Age effects on ASD symptomatology can be suspected, especially in the case of NF1, but longitudinal studies to corroborate this are missing. There is a lack of validated, uniform, and comprehensive in‐depth measurements of ASD traits in studies, inconsistent use of controls, and inconsistent choices of control groups. Although we have not found a clear answer to our research questions, an important added value of our review is that it not only summarizes what is already known, but critically reexamines the interpretation of some findings that are put forward in the research literature. This concerns, among other things, the question to what extent the different instruments measure the same construct and how specifically it concerns ASD‐related behavior instead of other developmental problems. In addition, we raise the question of how the results of scientific research relate to the clinical diagnoses of ASD in RASopathies, and why this has not received much attention so far. The added value of specifically delineating subgroups with a research diagnosis of ASD for separate analyses is also questioned in light of the continuous distribution of ASD characteristics. In addition to critically questioning some assumptions in the literature and identifying methodological shortcomings that hinder a better understanding, we also formulate several recommendations that can remedy this. We urge researchers to consider a dimensional approach across diagnostic boundaries. To get a grip on the ASD phenotype in RASopathies and advance the fields of behavioral genetics and developmental psychiatry, future research should use samples of adequate size and with molecularly confirmed clinical diagnoses, comprehensive phenotyping using reliable instruments, and control groups that are consistent with the research questions.

## AUTHOR CONTRIBUTIONS

E.D. conceptualized the research questions. M.E.B. performed the initial literature search and screened the records, after which E.D. independently validated these steps. E.D. and M.E.B. drafted the manuscript. J.S. critically revised the manuscript.

## ACKNOWLEDGEMENTS

The authors did not receive any financial or material support for conducting this review. Apart from the authors no other persons contributed.

## CONFLICT OF INTEREST STATEMENT

None.

## Supporting information


**Data S1.**.

## Data Availability

Data on the authors' own calculations are available from the corresponding author upon reasonable request. Otherwise, data sharing is not applicable to this article as no new data were created or analyzed in this study.

## References

[mgg32428-bib-0001] Adviento, B. , Corbin, I. L. , Widjaja, F. , Desachy, G. , Enrique, N. , Rosser, T. , Risi, S. , Marco, E. J. , Hendren, R. L. , Bearden, C. E. , Rauen, K. A. , & Weiss, L. A. (2014). Autism traits in the RASopathies. Journal of Medical Genetics, 51(1), 10–20. 10.1136/jmedgenet-2013-101951 24101678 PMC4230531

[mgg32428-bib-0002] Alfieri, P. , Piccini, G. , Caciolo, C. , Perrino, F. , Gambardella, M. L. , Mallardi, M. , Cesarini, L. , Leoni, C. , Leone, D. , Fossati, C. , Selicorni, A. , Digilio, M. C. , Tartaglia, M. , Mercuri, E. , Zampino, G. , & Vicari, S. (2014). Behavioral profile in RASopathies. American Journal of Medical Genetics Part A, 164(4), 934–942. 10.1002/ajmg.a.36374 24458522

[mgg32428-bib-0003] American Psychiatric Association . (1994). DSM‐IV: Diagnostic and statistical manual of mental disorders (4th ed.). American Psychiatric Association.

[mgg32428-bib-0004] American Psychiatric Association . (2013). Diagnostic and statistical manual of mental disorders (5th ed.). American Psychiatric Association Publishing. Retrieved May 31, 2018, from https://www.appi.org/Course/Book/Subscription/JournalSubscription/id‐3322/Diagnostic_and_Statistical_Manual_of_Mental_Disorders_%28DSM‐5®%29

[mgg32428-bib-0005] Axelrad, M. E. , Schwartz, D. D. , Katzenstein, J. M. , Hopkins, E. , & Gripp, K. W. (2011). Neurocognitive, adaptive, and behavioral functioning of individuals with Costello syndrome: A review. American Journal of Medical Genetics Part C, Seminars in Medical Genetics, 157(2), 115–122. 10.1002/ajmg.c.30299 21495179

[mgg32428-bib-0006] Berument, S. K. , Rutter, M. , Lord, C. , Pickles, A. , & Bailey, A. (1999). Autism screening questionnaire: Diagnostic validity. British Journal of Psychiatry, 175, 444–451. 10.1192/bjp.175.5.444 10789276

[mgg32428-bib-0007] Bessis, D. , Miquel, J. , Bourrat, E. , Chiaverini, C. , Morice‐Picard, F. , Abadie, C. , Manna, F. , Baumann, C. , Best, M. , Blanchet, P. , Bursztejn, A. C. , Capri, Y. , Coubes, C. , Giuliano, F. , Guillaumont, S. , Hadj‐Rabia, S. , Jacquemont, M. L. , Jeandel, C. , Lacombe, D. , … Cavé, H. (2019). Dermatological manifestations in Noonan syndrome: A prospective multicentric study of 129 patients positive for mutation. British Journal of Dermatology, 180(6), 1438–1448. 10.1111/BJD.17404 30417923

[mgg32428-bib-0008] Bilder, D. A. , Bakian, A. V. , Stevenson, D. A. , Carbone, P. S. , Cunniff, C. , Goodman, A. B. , McMahon, W. M. , Fisher, N. P. , & Viskochil, D. (2016). Brief report: The prevalence of neurofibromatosis type 1 among children with autism spectrum disorder identified by the autism and developmental disabilities monitoring network. Journal of Autism and Developmental Disorders, 46(10), 3369–3376. 10.1007/s10803-016-2877-3 27465244 PMC5494711

[mgg32428-bib-0009] Chisholm, A. K. , Anderson, V. A. , Pride, N. A. , Malarbi, S. , North, K. N. , & Payne, J. M. (2018a). Social function and autism spectrum disorder in children and adults with neurofibromatosis type 1: A systematic review and meta‐analysis. Neuropsychology Review, 28(3), 317–340. 10.1007/s11065-018-9380-x 30097761

[mgg32428-bib-0010] Chisholm, A. K. , Haebich, K. M. , Pride, N. A. , Walsh, K. S. , Lami, F. , Ure, A. , Maloof, T. , Brignell, A. , Rouel, M. , Granader, Y. , Maier, A. , Barton, B. , Darke, H. , Dabscheck, G. , Anderson, V. A. , Williams, K. , North, K. N. , & Payne, J. M. (2022). Delineating the autistic phenotype in children with neurofibromatosis type 1. Molecular Autism, 13(1), 3. 10.1186/s13229-021-00481-3 34983638 PMC8729013

[mgg32428-bib-0011] Chisholm, A. K. , Lami, F. , Haebich, K. M. , Ure, A. , Brignell, A. , Maloof, T. , Pride, N. A. , Walsh, K. S. , Maier, A. , Rouel, M. , Granader, Y. , Barton, B. , Darke, H. , Fuelscher, I. , Dabscheck, G. , Anderson, V. A. , Williams, K. , North, K. N. , & Payne, J. M. (2023). Sex‐ and age‐related differences in autistic behaviours in children with neurofibromatosis type 1. Journal of Autism and Developmental Disorders, 53(7), 2835–2850. 10.1007/s10803-022-05571-6 35445370

[mgg32428-bib-0012] Cohen, R. , Halevi, A. , Aharoni, S. , Aronson, B. , & Diamond, G. (2022). Impairments in communication and social interaction in children with neurofibromatosis type 1: Characteristics and role of ADHD and language delay. Applied Neuropsychology: Child, 11(3), 220–225. 10.1080/21622965.2020.1780924 32569512

[mgg32428-bib-0013] Constantino, J. N. (2002). The social responsiveness scale. In Encyclopedia of autism spectrum disorders (pp. 4457–4467). Springer International Publishing.

[mgg32428-bib-0014] Constantino, J. N. , Zhang, Y. , Holzhauer, K. , Sant, S. , Long, K. , Vallorani, A. , Malik, L. , & Gutmann, D. H. (2015). Distribution and within‐family specificity of quantitative autistic traits in patients with neurofibromatosis type I. The Journal of Pediatrics, 167(3), 621–626.e1. 10.1016/j.jpeds.2015.04.075 26051969 PMC4792262

[mgg32428-bib-0015] Eijk, S. , Mous, S. E. , Dieleman, G. C. , Dierckx, B. , Rietman, A. B. , de Nijs, P. F. A. , ten Hoopen, L. W. , van Minkelen, R. , Elgersma, Y. , Catsman‐Berrevoets, C. E. , Oostenbrink, R. , & Legerstee, J. S. (2018). Autism spectrum disorder in an unselected cohort of children with neurofibromatosis type 1 (NF1). Journal of Autism and Developmental Disorders, 48(7), 2278–2285. 10.1007/s10803-018-3478-0 29423604 PMC5995999

[mgg32428-bib-0016] Fombonne, E. , Morotti, H. , Mastel, S. , Keller, K. , Barnard, R. A. , Hall, T. , & O'Roak, B. J. (2021). Autism questionnaire scores do not only rise because of autism. Developmental Medicine and Child Neurology, 63(2), 235–236. 10.1111/DMCN.14725 33118173

[mgg32428-bib-0017] Foy, A. M. H. , Hudock, R. L. , Shanley, R. , & Pierpont, E. I. (2022). Social behavior in RASopathies and idiopathic autism. Journal of Neurodevelopmental Disorders, 14(1), 5. 10.1186/s11689-021-09414-w 35021989 PMC8753327

[mgg32428-bib-0018] Friedman, J. M. , Arbiser, J. , Epstein, J. A. , Gutmann, D. H. , Huot, S. J. , Lin, A. E. , Mcmanus, B. , & Korf, B. R. (2002). Cardiovascular disease in neurofibromatosis 1: Report of the NF1 cardiovascular task force. Genetics in Medicine, 4(3), 105–111. 10.1097/00125817-200205000-00002 12180143

[mgg32428-bib-0019] Garg, S. , Brooks, A. , Burns, A. , Burkitt‐Wright, E. , Kerr, B. , Huson, S. , Emsley, R. , & Green, J. (2017). Autism spectrum disorder and other neurobehavioural comorbidities in rare disorders of the Ras/MAPK pathway. Developmental Medicine and Child Neurology, 59(5), 544–549. 10.1111/DMCN.13394 28160302

[mgg32428-bib-0020] Garg, S. , Green, J. , Leadbitter, K. , Emsley, R. , Lehtonen, A. , Evans, D. G. , & Huson, S. M. (2013). Neurofibromatosis type 1 and autism spectrum disorder. Pediatrics, 132(6), e1642–e1648. Retrieved September 14, 2018, from. www.aappublications.org/news 24190681 10.1542/peds.2013-1868

[mgg32428-bib-0021] Garg, S. , Heuvelman, H. , Huson, S. , Tobin, H. , Green, J. , & Northern UK NF1 Research Network . (2016). Sex bias in autism spectrum disorder in neurofibromatosis type 1. Journal of Neurodevelopmental Disorders, 8, 26. 10.1186/s11689-016-9159-4 27516813 PMC4980803

[mgg32428-bib-0022] Garg, S. , Lehtonen, A. , Huson, S. M. , Emsley, R. , Trump, D. , Evans, D. G. , & Green, J. (2013). Autism and other psychiatric comorbidity in neurofibromatosis type 1: Evidence from a population‐based study. Developmental Medicine and Child Neurology, 55(2), 139–145. 10.1111/DMCN.12043 23163236

[mgg32428-bib-0023] Garg, S. , Plasschaert, E. , Descheemaeker, M. J. , Huson, S. , Borghgraef, M. , Vogels, A. , Evans, D. G. , Legius, E. , & Green, J. (2015). Autism spectrum disorder profile in neurofibromatosis type I. Journal of Autism and Developmental Disorders, 45(6), 1649–1657. 10.1007/s10803-014-2321-5 25475362

[mgg32428-bib-0024] Geoffray, M. M. , Falissard, B. , Green, J. , Kerr, B. , Evans, D. G. , Huson, S. , Burkitt‐Wright, E. , & Garg, S. (2021). Autism spectrum disorder symptom profile across the RASopathies. Frontiers in Psychiatry, 11, 585700. 10.3389/FPSYT.2020.585700/BIBTEX 33519543 PMC7843573

[mgg32428-bib-0025] Glad, D. M. , Casnar, C. L. , Yund, B. D. , Lee, K. , & Klein‐Tasman, B. P. (2021). Parent‐reported social skills in children with neurofibromatosis type 1: Longitudinal patterns and relations with attention and cognitive functioning. Journal of Developmental and Behavioral Pediatrics, 42(8), 656–665. 10.1097/DBP.0000000000000939 34618723 PMC8944791

[mgg32428-bib-0026] Grant, A. R. , Cushman, B. J. , Cavé, H. , Dillon, M. W. , Gelb, B. D. , Gripp, K. W. , Lee, J. A. , Mason‐Suares, H. , Rauen, K. A. , Tartaglia, M. , Vincent, L. M. , & Zenker, M. (2018). Assessing the gene–disease association of 19 genes with the RASopathies using the ClinGen gene curation framework. Human Mutation, 39(11), 1485–1493. 10.1002/humu.23624 30311384 PMC6326381

[mgg32428-bib-0027] Grzadzinski, R. , Dick, C. , Lord, C. , & Bishop, S. (2016). Parent‐reported and clinician‐observed autism spectrum disorder (ASD) symptoms in children with attention deficit/hyperactivity disorder (ADHD): Implications for practice under DSM‐5. Molecular Autism, 7(1), 1–12. 10.1186/s13229-016-0072-1 26788284 PMC4717584

[mgg32428-bib-0028] Haebich, K. M. , Dao, D. P. , Pride, N. A. , Barton, B. , Walsh, K. S. , Maier, A. , Chisholm, A. K. , Darke, H. , Catroppa, C. , Malarbi, S. , Wilkinson, J. C. , Anderson, V. A. , North, K. N. , & Payne, J. M. (2022). The mediating role of ADHD symptoms between executive function and social skills in children with neurofibromatosis type 1. Child Neuropsychology, 28(3), 318–336. 10.1080/09297049.2021.1976129 34587865

[mgg32428-bib-0029] Havdahl, K. A. , Hus Bal, V. , Huerta, M. , Pickles, A. , Øyen, A. S. , Stoltenberg, C. , Lord, C. , & Bishop, S. L. (2016). Multidimensional influences on autism symptom measures: Implications for use in etiological. Research, 55, 1054–1063.e3. www.jaacap.org 10.1016/j.jaac.2016.09.490 PMC5131801 27871640

[mgg32428-bib-0030] Hirabaru, K. , & Matsuo, M. (2018). Neurological comorbidity in children with neurofibromatosis type 1. Pediatrics International, 60(1), 70–75. 10.1111/ped.13388 28796925

[mgg32428-bib-0031] Huijbregts, S. C. J. , & de Sonneville, L. M. J. (2011). Does cognitive impairment explain behavioral and social problems of children with neurofibromatosis type 1? Behavior Genetics, 41(3), 430–436. 10.1007/s10519-010-9430-5 21184163 PMC3102189

[mgg32428-bib-0032] Hus Bal, V. , & Lord, C. (2015). Replication of standardized ADOS domain scores in the Simons simplex collection. Autism Research, 8(5), 583–592. 10.1002/AUR.1474 25712123 PMC4876493

[mgg32428-bib-0033] Hus, V. , Bishop, S. , Gotham, K. , Huerta, M. , & Lord, C. (2013). Factors influencing scores on the social responsiveness scale. Journal of Child Psychology and Psychiatry, 54(2), 216–224. 10.1111/j.1469-7610.2012.02589.x 22823182 PMC3504640

[mgg32428-bib-0034] Hus, V. , Gotham, K. , & Lord, C. (2014). Standardizing ADOS domain scores: Separating severity of social affect and restricted and repetitive behaviors. Journal of Autism and Developmental Disorders, 44(10), 2400–2412. 10.1007/s10803-012-1719-1 23143131 PMC3612387

[mgg32428-bib-0035] Jafry, M. , & Sidbury, R. (2020). RASopathies. Clinics in Dermatology, 38(4), 455–461. 10.1016/J.CLINDERMATOL.2020.03.010 32972603

[mgg32428-bib-0036] Janusz, J. A. , Klein‐Tasman, B. P. , Payne, J. M. , Wolters, P. L. , Thompson, H. L. , Martin, S. , de Blank, P. , Ullrich, N. , del Castillo, A. , Hussey, M. , Hardy, K. K. , Haebich, K. , Rosser, T. , Toledo‐Tamula, M. A. , Walsh, K. S. , & on behalf of the REiNS International Collaboration . (2021). Recommendations for social skills end points for clinical trials in neurofibromatosis type 1. Neurology, 97(7), S73–S80. 10.1212/WNL.0000000000012422 34230205 PMC8594002

[mgg32428-bib-0037] Kehrer‐Sawatzki, H. , Kluwe, L. , Salamon, J. , Well, L. , Farschtschi, S. , Rosenbaum, T. , & Mautner, V. F. (2020). Clinical characterization of children and adolescents with NF1 microdeletions. Child's Nervous System, 36(10), 2297–2310. 10.1007/s00381-020-04717-0 PMC7575500 32533297

[mgg32428-bib-0038] Kenborg, L. , Andersen, E. W. , Duun‐Henriksen, A. K. , Jepsen, J. R. M. , Doser, K. , Dalton, S. O. , Bidstrup, P. E. , Krøyer, A. , Frederiksen, L. E. , Johansen, C. , Østergaard, J. R. , Hove, H. , Sørensen, S. A. , Riccardi, V. M. , Mulvihill, J. J. , & Winther, J. F. (2021). Psychiatric disorders in individuals with neurofibromatosis 1 in Denmark: A nationwide register‐based cohort study. American Journal of Medical Genetics Part A, 185(12), 3706–3716. 10.1002/ajmg.a.62436 34327813

[mgg32428-bib-0039] King, B. H. , Navot, N. , Bernier, R. , & Webb, S. J. (2014). Update on diagnostic classification in autism. Current Opinion in Psychiatry, 27(2), 105–109. 10.1097/YCO.0000000000000040 24441420 PMC4929984

[mgg32428-bib-0040] Lainhart, J. E. , Bigler, E. D. , Bocian, M. , Coon, H. , Dinh, E. , Dawson, G. , Deutsch, C. K. , Dunn, M. , Estes, A. , Tager‐Flusberg, H. , Folstein, S. , Hepburn, S. , Hyman, S. , McMahon, W. , Minshew, N. , Munson, J. , Osann, K. , Ozonoff, S. , Rodier, P. , … Volkmar, F. (2006). Head circumference and height in autism: A study by the collaborative program of excellence in autism. American Journal of Medical Genetics Part A, 140A(21), 2257–2274. 10.1002/AJMG.A.31465 PMC4899843 17022081

[mgg32428-bib-0041] Lalancette, E. , Charlebois‐Poirier, A. R. , Agbogba, K. , Knoth, I. S. , Jones, E. J. H. , Mason, L. , Perreault, S. , & Lippé, S. (2022). Steady‐state visual evoked potentials in children with neurofibromatosis type 1: Associations with behavioral rating scales and impact of psychostimulant medication. Journal of Neurodevelopmental Disorders, 14(1), 42. 10.1186/s11689-022-09452-y 35869419 PMC9306184

[mgg32428-bib-0042] Legius, E. , Messiaen, L. , Wolkenstein, P. , Pancza, P. , Avery, R. A. , Berman, Y. , Blakeley, J. , Babovic‐Vuksanovic, D. , Cunha, K. S. , Ferner, R. , Fisher, M. J. , Friedman, J. M. , Gutmann, D. H. , Kehrer‐Sawatzki, H. , Korf, B. R. , Mautner, V. F. , Peltonen, S. , Rauen, K. A. , Riccardi, V. , … Plotkin, S. R. (2021). Revised diagnostic criteria for neurofibromatosis type 1 and Legius syndrome: An international consensus recommendation. Genetics in Medicine, 23(8), 1506–1513. 10.1038/S41436-021-01170-5/ATTACHMENT/FC55EA50-5CA2-4891-BE65-FE6F6C9BADAE/MMC1.PDF 34012067 PMC8354850

[mgg32428-bib-0043] Loitfelder, M. , Huijbregts, S. C. J. , Veer, I. M. , Swaab, H. S. , van Buchem, M. A. , Schmidt, R. , & Rombouts, S. A. (2015). Functional connectivity changes and executive and social problems in neurofibromatosis type I. Brain Connectivity, 5(5), 320. 10.1089/BRAIN.2014.0334 PMC4490703 25705926

[mgg32428-bib-0044] Loomes, R. , Hull, L. , & Mandy, W. P. L. (2017). What is the male‐to‐female ratio in autism spectrum disorder? A systematic review and meta‐analysis. Journal of the American Academy of Child and Adolescent Psychiatry, 56(6), 466–474. 10.1016/J.JAAC.2017.03.013 28545751

[mgg32428-bib-0045] Lord, C. , Rutter, M. , Dilavore, P. C. , Risi, S. , Gotham, K. , & Bishop, S. L. (2012). Autism diagnostic observation schedule (2nd ed.). Western Psychological Services.

[mgg32428-bib-0046] Lubbers, K. , Stijl, E. M. , Dierckx, B. , Hagenaar, D. A. , ten Hoopen, L. W. , Legerstee, J. S. , de Nijs, P. F. A. , Rietman, A. B. , Greaves‐Lord, K. , Hillegers, M. H. J. , Dieleman, G. C. , Mous, S. E. , & ENCORE Expertise Center . (2022). Autism symptoms in children and young adults with fragile X syndrome, Angelman syndrome, tuberous sclerosis complex, and neurofibromatosis type 1: A cross‐syndrome comparison. Frontiers in Psychiatry, 13, 852208. 10.3389/fpsyt.2022.852208 35651825 PMC9149157

[mgg32428-bib-0047] McCubrey, J. A. , Steelman, L. S. , Chappell, W. H. , Abrams, S. L. , Wong, E. W. , Chang, F. , Lehmann, B. , Terrian, D. M. , Milella, M. , Tafuri, A. , & Stivala, F. (2007). Roles of the Raf/MEK/ERK pathway in cell growth, malignant transformation and drug resistance. Biochimica et Biophysica Acta (BBA)‐Molecular Cell Research, 1773(8), 1263–1284. 10.1016/J.BBAMCR.2006.10.001 17126425 PMC2696318

[mgg32428-bib-0048] Morotti, H. , Mastel, S. , Keller, K. , Barnard, R. A. , Hall, T. , O'Roak, B. J. , & Fombonne, E. (2021). Autism and attention‐deficit/hyperactivity disorders and symptoms in children with neurofibromatosis type 1. Developmental Medicine and Child Neurology, 63(2), 226–232. 10.1111/dmcn.14558 32406525

[mgg32428-bib-0049] Morris, S. M. , Acosta, M. T. , Garg, S. , Green, J. , Huson, S. , Legius, E. , North, K. N. , Payne, J. M. , Plasschaert, E. , Frazier, T. W. , Weiss, L. A. , Zhang, Y. , Gutmann, D. H. , & Constantino, J. N. (2016). Disease burden and symptom structure of autism in neurofibromatosis type 1. A study of the international NF1‐ASD consortium team (INFACT). JAMA Psychiatry, 73(12), 1276–1284. 10.1001/jamapsychiatry.2016.2600 27760236 PMC5298203

[mgg32428-bib-0050] Morris, S. M. , Acosta, M. T. , Garg, S. , Green, J. , Legius, E. , North, K. , Payne, J. M. , Weiss, L. A. , Constantino, J. N. , & Gutmann, D. H. (2021). Autism in neurofibromatosis type 1: Misuse of covariance to dismiss autistic trait burden. Developmental Medicine and Child Neurology, 63(2), 233–234. 10.1111/dmcn.14653 32815557

[mgg32428-bib-0051] Morris, S. M. , & Gutmann, D. H. (2018). A genotype–phenotype correlation for quantitative autistic trait burden in neurofibromatosis 1. Neurology, 90(8), 377–379. 10.1212/WNL.0000000000005000 29367450 PMC10681069

[mgg32428-bib-0052] Muram‐Zborovski, T. M. , Stevenson, D. A. , Viskochil, D. H. , Dries, D. C. , Wilson, A. R. , & Mao, R. (2010). SPRED 1 mutations in a neurofibromatosis clinic. Journal of Child Neurology, 25(10), 1203–1209. 10.1177/0883073809359540 20179001 PMC3243064

[mgg32428-bib-0053] National Institutes of Health . (1988). Consensus development conference: Neurofibromatosis conference statement. Archives of Neurology, 45(5), 575–578. 10.1001/ARCHNEUR.1988.00520290115023 3128965

[mgg32428-bib-0054] Naylor, P. E. , Bruno, J. L. , Shrestha, S. B. , Friedman, M. , Jo, B. , Reiss, A. L. , & Green, T. (2023). Neuropsychiatric phenotypes in children with Noonan syndrome. Developmental Medicine and Child Neurology, 65(11), 1520–1529. 10.1111/dmcn.15627 37130201 PMC10592553

[mgg32428-bib-0055] Niemczyk, J. , Equit, M. , Borggrefe‐Moussavian, S. , Curfs, L. , & von Gontard, A. (2015). Incontinence in persons with Noonan syndrome. Journal of Pediatric Urology, 11(4), 201.e1–201.e5. 10.1016/J.JPUROL.2015.06.002 26143485

[mgg32428-bib-0056] Onesimo, R. , Sforza, E. , Giorgio, V. , Viscogliosi, G. , Kuczynska, E. M. , Margiotta, G. , Perri, L. , Limongelli, D. , Proli, F. , de Rose, C. , Rigante, D. , Cerchiari, A. , Tartaglia, M. , Leoni, C. , & Zampino, G. (2023). The “FEEDS (FEeding eating deglutition skills)” over time study in Cardiofaciocutaneous syndrome. Genes (Basel), 14(7), 1338. 10.3390/genes14071338 37510243 PMC10379052

[mgg32428-bib-0057] Page, M. J. , McKenzie, J. E. , Bossuyt, P. M. , Boutron, I. , Hoffmann, T. C. , Mulrow, C. D. , Shamseer, L. , Tetzlaff, J. M. , Akl, E. A. , Brennan, S. E. , Chou, R. , Glanville, J. , Grimshaw, J. M. , Hróbjartsson, A. , Lalu, M. M. , Li, T. , Loder, E. W. , Mayo‐Wilson, E. , McDonald, S. , … Moher, D. (2021). The PRISMA 2020 statement: An updated guideline for reporting systematic reviews. BMJ, 372, n71. 10.1136/BMJ.N71 33782057 PMC8005924

[mgg32428-bib-0058] Payne, J. M. , Walsh, K. S. , Pride, N. A. , Haebich, K. M. , Maier, A. , Chisholm, A. , Glad, D. M. , Casnar, C. L. , Rouel, M. , Lorenzo, J. , Del Castillo, A. , North, K. N. , & Klein‐Tasman, B. (2020). Social skills and autism spectrum disorder symptoms in children with neurofibromatosis type 1: Evidence for clinical trial outcomes. Developmental Medicine and Child Neurology, 62(7), 813–819. 10.1111/DMCN.14517/ABSTRACT 32181506

[mgg32428-bib-0059] Pierpont, E. I. , Hudock, R. L. , Foy, A. M. , Semrud‐Clikeman, M. , Pierpont, M. E. , Berry, S. A. , Shanley, R. , Rubin, N. , Sommer, K. , & Moertel, C. L. (2018). Social skills in children with RASopathies: A comparison of Noonan syndrome and neurofibromatosis type 1. Journal of Neurodevelopmental Disorders, 10(1), 21. 10.1186/s11689-018-9239-8 29914349 PMC6006579

[mgg32428-bib-0060] Pierpont, M. E. M. , Magoulas, P. L. , Adi, S. , Kavamura, M. I. , Neri, G. , Noonan, J. , Pierpont, E. I. , Reinker, K. , Roberts, A. E. , Shankar, S. , Sullivan, J. , Wolford, M. , Conger, B. , Santa Cruz, M. , & Rauen, K. A. (2014). Cardio‐Facio‐cutaneous syndrome: Clinical features, diagnosis, and management guidelines. Pediatrics, 134, 1149–1162. http://publications.aap.org/pediatrics/article‐pdf/134/4/e1149/1099591/peds_2013‐3189.pdf?casa_token=mPSF7Rgfpl0AAAAA:9MUGnzVKCpf‐z81EzdHTC‐ttgOPuImfitkhjXEqQ‐DTQ0vTqau3ZKW‐nv1D 10.1542/peds.2013-3189 PMC4179092 25180280

[mgg32428-bib-0061] Plasschaert, E. , Descheemaeker, M. J. , Van Eylen, L. , Noens, I. , Steyaert, J. , & Legius, E. (2015). Prevalence of autism spectrum disorder symptoms in children with neurofibromatosis type 1. American Journal of Medical Genetics Part B, Neuropsychiatric Genetics, 168B(1), 72–80. 10.1002/ajmg.b.32280 25388972

[mgg32428-bib-0062] Plasschaert, E. , Van Eylen, L. , Descheemaeker, M. J. , Noens, I. , Legius, E. , & Steyaert, J. (2016). Executive functioning deficits in children with neurofibromatosis type 1: The influence of intellectual and social functioning. American Journal of Medical Genetics Part B, Neuropsychiatric Genetics, 171B(3), 348–362. 10.1002/ajmg.b.32414 26773288

[mgg32428-bib-0063] Pride, N. A. , Crawford, H. , Payne, J. M. , & North, K. N. (2013). Social functioning in adults with neurofibromatosis type 1. Research in Developmental Disabilities, 34(10), 3393–3399. 10.1016/j.ridd.2013.07.011 23911645

[mgg32428-bib-0064] Pride, N. A. , Haebich, K. M. , Walsh, K. S. , Lami, F. , Rouel, M. , Maier, A. , Chisholm, A. K. , Lorenzo, J. , Hearps, S. J. C. , North, K. N. , & Payne, J. M. (2023). Sensory processing in children and adolescents with neurofibromatosis type 1. Cancers (Basel), 15(14), 3612. 10.3390/cancers15143612 37509275 PMC10377664

[mgg32428-bib-0065] Rauen, K. A. (2022). Defining RASopathy. Disease Models & Mechanisms, 15(2), dmm04934. 10.1242/dmm.049344 PMC8821523 35103797

[mgg32428-bib-0066] Rauen, K. A. , Schoyer, L. , Schill, L. , Stronach, B. , Albeck, J. , Andresen, B. S. , Cavé, H. , Ellis, M. , Fruchtman, S. M. , Gelb, B. D. , Gibson, C. C. , Gripp, K. , Hefner, E. , Huang, W. Y. C. , Itkin, M. , Kerr, B. , Linardic, C. M. , McMahon, M. , Oberlander, B. , … McCormick, F. (2018). Proceedings of the fifth international RASopathies symposium: When development and cancer intersect. American Journal of Medical Genetics Part A, 176(12), 2924–2929. 10.1002/AJMG.A.40632 30302932 PMC6312476

[mgg32428-bib-0067] Rethlefsen, M. L. , Kirtley, S. , Waffenschmidt, S. , Ayala, A. P. , Moher, D. , Page, M. J. , Koffel, J. B. , & Group, P. R. I. S. M. A. S. (2021). PRISMA‐S: An extension to the PRISMA statement for reporting literature searches in systematic reviews. Journal of the Medical Library Association, 109(2), 200. 10.5195/JMLA.2021.962 PMC8270366 34285662

[mgg32428-bib-0068] Robins, D. L. , Fein, D. , Barton, M. L. , & Green, J. A. (2001). The modified checklist for autism in toddlers: An initial study investigating the early detection of autism and pervasive developmental disorders. Journal of Autism and Developmental Disorders, 31(2), 131–144. 11450812 10.1023/a:1010738829569

[mgg32428-bib-0069] Rutter, M. , LeCouteur, A. , & Lord, C. (2003). Autism diagnostic interview‐revised. Western Psychological Services.

[mgg32428-bib-0070] Schwartz, D. D. , Katzenstein, J. M. , Highley, E. J. , Stabley, D. L. , Sol‐Church, K. , Gripp, K. W. , & Axelrad, M. E. (2017). Age‐related differences in prevalence of autism spectrum disorder symptoms in children and adolescents with Costello syndrome. American Journal of Medical Genetics Part A, 173(5), 1294–1300. 10.1002/AJMG.A.38174 28374929 PMC5397350

[mgg32428-bib-0071] Siegel, D. H. , Mann, J. A. , Krol, A. L. , & Rauen, K. A. (2012). Dermatological phenotype in Costello syndrome: Consequences of Ras dysregulation in development. British Journal of Dermatology, 166(3), 601–607. 10.1111/J.1365-2133.2011.10744.X 22098123 PMC4063554

[mgg32428-bib-0072] Siegel, D. H. , McKenzie, J. , Frieden, I. J. , & Rauen, K. A. (2011). Dermatological findings in 61 mutation‐positive individuals with cardiofaciocutaneous syndrome. British Journal of Dermatology, 164(3), 521–529. 10.1111/J.1365-2133.2010.10122.X 21062266 PMC4063552

[mgg32428-bib-0073] Stivaros, S. , Garg, S. , Tziraki, M. , Cai, Y. , Thomas, O. , Mellor, J. , Morris, A. A. , Jim, C. , Szumanska‐Ryt, K. , Parkes, L. M. , Haroon, H. A. , Montaldi, D. , Webb, N. , Keane, J. , Castellanos, F. X. , Silva, A. J. , Huson, S. , Williams, S. , Gareth Evans, D. , … Green, J. (2018). Randomised controlled trial of simvastatin treatment for autism in young children with neurofibromatosis type 1 (SANTA). Molecular Autism, 9, 12. 10.1186/S13229-018-0190-Z/TABLES/1 29484149 PMC5824534

[mgg32428-bib-0074] Tidyman, W. E. , & Rauen, K. A. (2016). Pathogenetics of the RASopathies. Human Molecular Genetics, 25(R2), R123–R132. 10.1093/HMG/DDW191 27412009 PMC6283265

[mgg32428-bib-0075] Tinker, J. , Carbone, P. S. , Viskochil, D. , Mathiesen, A. , Ma, K. N. , & Stevenson, D. A. (2014). Screening children with neurofibromatosis type 1 for autism spectrum disorder. American Journal of Medical Genetics Part A, 164A(7), 1706–1712. 10.1002/ajmg.a.36549 24715629

[mgg32428-bib-0076] van Eeghen, A. M. , Pulsifer, M. B. , Merker, V. L. , Neumeyer, A. M. , van Eeghen, E. E. , Thibert, R. L. , Cole, A. J. , Leigh, F. A. , Plotkin, S. R. , & Thiele, E. A. (2013). Understanding relationships between autism, intelligence, and epilepsy: A cross‐disorder approach. Developmental Medicine and Child Neurology, 55(2), 146–153. 10.1111/DMCN.12044 23205844 PMC4071146

[mgg32428-bib-0077] Vandenbroucke, J. P. , von Elm, E. , Altman, D. G. , Gøtzsche, P. C. , Mulrow, C. D. , Pocock, S. J. , Poole, C. , Schlesselman, J. J. , Egger, M. , & for the STROBE Initiative . (2007). Strengthening the Reporting of Observational Studies in Epidemiology (STROBE): Explanation and elaboration. PLoS Medicine, 4(10), e297. 10.1371/journal.pmed.0040297 17941715 PMC2020496

[mgg32428-bib-0078] Viskochil, D. , Buchberg, A. M. , Xu, G. , Cawthon, R. M. , Stevens, J. , Wolff, R. K. , Culver, M. , Carey, J. C. , Copeland, N. G. , Jenkins, N. A. , White, R. , & O'Connell, P. (1990). Deletions and a translocation interrupt a cloned gene at the neurofibromatosis type 1 locus. Cell, 62(1), 187–192. 10.1016/0092-8674(90)90252-A 1694727

[mgg32428-bib-0079] Von Elm, E. , Altman, D. G. , Egger, M. , Pocock, S. J. , Gøtzsche, P. C. , & Vandenbroucke, J. P. (2007). The Strengthening the Reporting of Observational Studies in Epidemiology (STROBE) statement: Guidelines for reporting observational studies. The Lancet, 370, 1453–1457. 10.1016/S0140-6736(07)61602-X 18064739

[mgg32428-bib-0080] Walsh, K. S. , Vélez, J. I. , Kardel, P. G. , Imas, D. M. , Muenke, M. , Packer, R. J. , Castellanos, F. X. , & Acosta, M. T. (2013). Symptomatology of autism spectrum disorder in a population with neurofibromatosis type 1. Developmental Medicine and Child Neurology, 55(2), 131–138. 10.1111/dmcn.12038 23163951

[mgg32428-bib-0081] Young, O. , Perati, S. , Weiss, L. A. , & Rauen, K. A. (2018). Age and ASD symptoms in Costello syndrome. American Journal of Medical Genetics Part A, 176(4), 1027–1028. 10.1002/ajmg.a.38641 29575620 PMC6011828

