# Supplementary material for: Autism spectrum disorder profiles in RASopathies: A systematic review
Source: Mol Genet Genomic Med. 2024 Apr 5;12(4):e2428. doi: 10.1002/mgg3.2428 (PMC10997847; doi:10.1002/mgg3.2428)
Supplement: Supplementary file 1 — Data S1.. [file MGG3-12-e2428-s001.docx]

**General remark:** Reference numbers in these Supplementary materials correspond to the Supplementary references at the end of this document. Reference numbers in the main manuscript correspond to the References section at the end of the main manuscript.

**Supplement 1: Search Sequence using PubMed, Web of Science and Open Grey, eligibility criteria, and inclusion/exclusion process**

The **search sequence** **used in PubMed** was:

("Autism Spectrum Disorder"[Mesh] OR learning disabilities OR autism OR autism traits OR autistic traits OR ADOS OR behavioural profile OR personality profile OR social skills OR social problems OR "Developmental Disabilities"[Mesh] OR developmental disorder) AND (RASopath* OR RAS/MAPK OR "Noonan Syndrome"[Mesh] OR "Neurofibromatosis 1"[Mesh] OR "Costello Syndrome"[Mesh] OR "Cardio-facio-cutaneous syndrome" [Supplementary Concept] OR "Legius syndrome" [Supplementary Concept] OR "LEOPARD Syndrome"[Mesh]).

The **search sequence used in the Web of Science Core Collection** was:

(DOP=(1994-01-01/2023-11-28)) AND (LA=(English)) AND (ALL=((Autism spectrum disorder) OR (Learning disabilities) OR (autism) OR (autism traits) OR (autistic traits) OR (ADOS) OR (behavioural profile) OR (personality profile) OR (social skills) OR (social problems) OR (developmental disabilities) OR (developmental disorder))) AND (ALL=((RASopath*) OR (“RAS/MAPK”) OR (Noonan syndrome) OR (neurofibromatosis type 1) OR (Costello syndrome) OR (Cardio-facio-cutaneous syndrome) OR (Legius syndrome) OR (LEOPARD syndrome))) This search sequence was slightly modified compared to the one used in PubMed because of the absence of Mesh terms.

The search sequences used in Open Grey was

("Autism spectrum disorder" OR "Learning disabilities" OR autism OR "autism traits" OR "autistic traits" OR ADOS OR "behavioural profile" OR "personality profile" OR "social skills" OR "social problems" OR "developmental disabilities" OR "developmental disorder") AND (RASopath* OR “RAS/MAPK” OR "Noonan syndrome" OR "neurofibromatosis type 1" OR "Costello syndrome" OR "Cardio-facio-cutaneous syndrome" OR "Legius syndrome" OR "LEOPARD syndrome")

The search was last carried out on November 28^th^ 2023. In the PubMed search engine we applied automatic filters, in line with our in- and exclusion criteria: We limited the publication date to starting from January 1^st^ 1994 using Custom range, set Species to Humans and Article language to English. The same filters were already incorporated in the search sequence for Web of Science.

**Results of the search and inclusion process**The initial literature search by MEB yielded 1986 records. After removing duplicate records (n=244), title and abstract of 1742 records were screened, based on which another 1610 records were excluded. Out of the remaining 132 records 131 full text articles were retrieved. After screening of the full text 96 articles were excluded, resulting in 35 articles included from the database search. After a second author (ED) independently performed the same search and selection process, his results were compared to those of MEB, and a consensus was reached. Two additional articles were included^1,2^, and one article that was initially included by MEB was excluded^3^. 47 records were identified through citation searching. Of these articles, 42 were excluded. This resulted in 36 articles included through database searching, 5 articles included through citation searching, and a total of 41 articles included in our systematic review.

**Supplement 2: Supplementary tables**

### TABLE S1: Characteristics of included studies

| **Authors and year** | **Study Design** | **Participants** | **ASD instruments** | **Other instruments** | **Molecular confirmation** | **Geographical location** |
| --- | --- | --- | --- | --- | --- | --- |
| Huijbregts et al., 2011^4^ | Cross-sectional study | NF1 (n=30), HC (n=30)  6-17 y | SRS | SSRS, SDQ, ANT, MS2D, ROA | unknown | Netherlands |
| ^†^Garg, Green, et al., 2013^5^ | Cross-sectional study | NF1 (n=47)  7-18 y | SRS, ADI-R, ADOS-G | VABS-II, WASI, Conners | unknown | UK |
| Garg, Lehtonen et al., 2013^6^ | Cross-sectional study | NF1 (n=109) 4-16 y | SRS | CPRS-R | unknown | UK |
| van Eeghen et al., 2013^7^ | Cross-sectional study | NF1 (n=50), TSC (n=64), EUC (n=66), iASD (n=210), US-ASD (n=130)  4-63 y | SRS | IQ | unknown | USA |
| Walsh et al., 2013^8^ | Cross-sectional study | NF1(n=66)  4-18 y | SRS | VADPRS | unknown | USA |
| Adviento et al., 2014^9^ | Cross-sectional study | NF1 (n=81), NS (n=52), CFC (n=54), CS (n=44), iASD (n=171), US (n=119)  10-45 y | SCQ, SRS, ADOS, ADI-R | VABS-II, WAIS/WISC | NF1: 11%  NS: 56%  CS: 84%  CFC: 83% | USA |
| Alfieri et al., 2014^10^ | Cross-sectional study | NS (n=38), CFC (n=11), CS (n=11), NS-ML (n=6), NS-LAH (n=4)   2-28 y | M-CHAT,  DSM-IV | CBCL, WAIS-R, WISC, VRB, GMDS | 100% | Italy |
| Tinker et al., 2014^11^ | Cross-sectional study | NF1 (n=67)  16 m-11 y | M-CHAT, CAST | / | unknown | USA |
| Constantino et al., 2015^12^ | Cross-sectional study | NF1 (n= 103)  3-77 y | SRS | Conners | unknown | USA |
| ^‡^Garg et al., 2015^13^ | Cross-sectional study | NF1+ASD (n=36)  4-16 y | SRS, ADOS2 | WASI | unknown | Belgium, UK |
| Loitfelder et al., 2015^14^ | Cross-sectional study | NF1 (n=14), HC (n=30)  <18 y | SRS | WISC, SSRS, CBCL, BRIEF | unknown | Netherlands |
| Niemczyk et al., 2015^15^ | Cross-sectional study | NS (n=29)  5-48 y | DBC-P, DBC-A | / | unknown | Germany |
| Plasschaert et al., 2015^16^ | Cross-sectional study | NF1 (n=82)  5-17 y | CSBQ, SRS | / | unknown | Belgium |
| Bilder et al., 2016^17^ | Cross-sectional study | ASD+NF1(n=22), ASD-NF1 (n= 12249) | DSM-IV-TR | / | unknown | USA |
| ^§^Garg et al., 2016^18^ | Cross-sectional study | NF1 (n=194)  4-18y | SRS, ADI-R, ADOS | Conners, WASI | unknown | UK |
| ^¶^Morris et al., 2016^19^ | Cross-sectional study | NF1 (n=531)  2- 83 y | SRS | Conners | unknown | Australia, Belgium, UK, USA |
| Plasschaert et al., 2016^20^ | Cross-sectional study | NF1 (n= 42), HC (n=52), iASD (n=52)  8-18 y | SRS | BRIEF, WISC/WAIS, EF and control measures | unknown | Belgium |
| Garg et al., 2017^21^ | Cross-sectional study | NS (n=40), CFC (n=9)  6-16 y | ADOS, ADI-R, DANVA | WASI-II, BRIEF, Conners, TEA-Ch, TOF | CFC: 100%  NS: 0% | UK |
| Schwartz et al., 2017^22^ | Cross-sectional study | CS (n=14)  1-18 y | M-CHAT, SCQ | VABS-II, Leiter-R | 100% | USA |
| Eijk et al., 2018^23^ | Cross-sectional study | NF1 (n=128)  2-10y | SRS, ADOS-G, ADOS-2, DSM-IV | WPPSI-III, WISC-III | At least 58.6% | Netherlands |
| Hirabaru et al., 2018^24^ | Cross-sectional study | NF1 (n=143)  3-15 y | SRS | ADHD-RS-IV | unknown | Japan |
| °Morris et al., 2018^25^ | Cross-sectional study | NF1(n=63)  2-58 y | SRS | / | 100% | USA |
| Pierpont et al. 2018^26^ | Cross-sectional study | NF1 (n=39), NS (n = 39), US (n=32)  8-16 y | SSIS | CCC-2, ADHD-RS, BASC2 | NS: 88%  NF1: 65% | USA |
| Stivaros et al., 2018^27^ | Randomised controlled trial | NF1+ASD (n=30)   4-10 y | ADI-R, ADOS-2, SRS | WASI, ABC, CGI-S, Conners | 90% | UK |
| ^#^Young et al., 2018^1^ | Cross-sectional study | CS (61≥n≥53) 1-32 y | SCQ, SRS | / | unknown | USA |
| Kehrer-Sawatzki et al., 2020^2^ | Cross-sectional study | NF1md (n=30) NF1iv (n=30) | SRS | MFED, HAWIK-R, HAWIK-III, HAWIK-IV | 100% | Germany |
| Payne et al., 2020^28^ | Cross-sectional study | NF1(n=122)  3-15 y | SRS, SSIS-RS | Conners | unknown | USA, Australia |
| ^£^Geoffray et al., 2021^29^ | Cross-sectional study | NF1+ASD (n=48),  NS+ASD (n=11),  CFC+ASD (n=7)  4-18 y | ADOS-2, ADI-R | WASI-II, Conners | NS: 8/11  NF1: unknown  CFC: 6/7 | UK |
| Morotti et al., 2021^30^ | Cross-sectional study | NF1 (n=45), iASD (n=180), US-ASD (n=180)  5-12 y | SRS, SCQ, RBS-R | CBCL, VABS-II | unknown | USA |
| Glad et al., 2021^31^ | Longitudinal | NF1 (n=65)  3-13 years | SSRS, SSIS | Conners, DAS-II | Unknown | USA |
| Kenborg et al., 2021^32^ | Cross-sectional | NF1 (n=905), non-NF1 (n=7614)  0-32 years | ICD code in DNPR |  | Unknown | Denmark |
| ^¥^Chisholm et al., 2022^33^ | Cross-sectional | NF1+ASD (n=68)  3-15 years | SRS, ADOS-2, ADI-R | WPPSI-IV, WISC-V, CADS, Conners, CBCL | Unknown | Australia, USA |
| Cohen et al., 2022^34^ | Cross-sectional | NF1-ASD (n=30)  5-18 years | SCQ | VABS II, DSM-IV-TR | Unknown | Israel |
| Foy et al., 2022^35^ | Cross-sectional | NF1 (n=70), NS (n=70), CS (n=15), CFC (n=25), iASD (n=97)  3-17 years | SEARS-P | SDQ, GO4KIDDS | NF1 69% NS 87% CS 100% CFC 97% | Australia, Canada, New Zealand, USA |
| ^¥^Haebich et al., 2022^36^ | Cross-sectional | NF1 (n=136), HC (n=93)  3-15 years | SSIS | WISC-V, WPPSI-IV, BRIEF, Conners | Unknown | Australia, USA |
| Lalancette et al., 2022^37^ | Cross-sectional | NF1 (n=28), HC (n=28)  4-13 years | SRS | IQ, Conners, CBCL | Unknown | Canada |
| Lubbers et al., 2022^38^ | Cross-sectional | NF1 (n=279), FXS (n=60), TSC (n=110), AS (n=91), iASD (n=206)  1-18 years | SRS, ADOS | WPPSI-III, WISC-III, WISC-V, WAIS-III, WNV, Bayley-III, SON | Unknown | Netherlands |
| ^¥^Chisholm et al., 2023^39^ | Cross-sectional | NF1 (n=62)  3-16 years | SRS, ADOS-2, ADI-R | CBCL, CADS, Conners, WPPSI-IV, WISC-V, CELF-Preschool-2, NEPSY-II, CELF-4 | Unknown | Australia, USA |
| Naylor et al., 2023^40^ | Cross-sectional | NS (n=45), HC (n=40)  4-11 years | KSADS, SRS | BASC-2, BRIEF, NEPSY-II, WPPSI-III, WISC-IV | 100% | USA |
| Onesimo et al., 2023^41^ | Cross-sectional | CFC (n=27)  1-38 years | SP | DQ5, I-MCH-FS | 100% | Italy |
| ^¥^Pride et al., 2023^42^ | Cross-sectional | NF1 (n=152); HC (n=96)  3-15 years | SRS, SSIS, SP | Conners, ABAS-3, CBCL, WPPSI-IV, WISC-V | Unknown | Australia, USA |

^†^ Examined a subset of participants from Garg, Lehtonen et al., 2013, stratified according to SRS T-scores ^‡^ Pooled NF1+ASD data from the samples of Garg, Green, et al., 2013, Plasschaert et al., 2015, and Stivaros et al., 2018. ^§^ Pooled data from Garg, Green et al., 2013, and Stivaros et al., 2018. ^¶^ Pooled original data with those from Garg, Lehtonen, et al., 2013, Walsh et al., 2013, Adviento et al., 2014, Constantino et al., 2015, and Plasschaert et al., 2015. ° Analyzed a subset of data from Constantino et al., 2015. ^#^ Pooled original data with those from Adviento et al., 2014 ^£^ Pooled a subset of data of NF1+ASD, CFC+ASD and CS+ASD from Garg, Green et al., 2013, Garg et al., 2017, and Stivaros et al., 2018. ^¥^ Chisholm et al., 2022, Haebich et al., 2022, Chisholm et al., 2023 and Pride et al., 2023 report data from overlapping samples from the same overarching study.

**ABAS-3** Adaptive Behavior Assessment System, 3^rd^ edition**, ABC** Aberrant Behaviour Checklist, **ADHD-RS** Attention Deficit Hyperactivity Disorder – Rating Scale, **ADI-R** Autism Diagnostic Interview-Revised, **ADOS** Autism Diagnostic Observation Schedule, **ADOS-G** Autism Diagnostic Observation Scale Generic, **ANT** Amsterdam Neuropsychological Tasks, **BASC-2** Behaviour Assessment System for Children: Second Edition, **Bayley-III** Bayley Scales of Infant and Toddler Development-3^rd^ edition, **BRIEF(-A)** Behaviour Rating Inventory of Executive Function(-Adult version), **CAST** Childhood Autism Spectrum Test, **CBCL** child behavior checklist, **CCC-2** Children’s Communication Checklist-2, **CELF-Preschool-2** Clinical Evaluation of Language Fundamentals – Preschool- 2^nd^ edition, **CELF-4** Clinical Evaluation of Language Fundamentals – 4^th^ edition, **CFC** cardio-facio-cutaneous syndrome, **CGI-S** Clinical Global Impression-Severity, **Conners** any Conners questionnaire rating ADHD symptoms, **CS** Costello syndrome, **CSBQ** Children’s Social Behaviour Questionnaire, **DANVA** Diagnostic Analysis of Nonverbal Accuracy, **DBC-A** Developmental Behaviour Checklist-Adult, **DBC-P** Developmental Behaviour Checklist- Parent, **DNPR** Danish National Patient Registry, **DQ5** Drooling Quotient 5, **DSM-IV(-TR)** Diagnostic and Statistical Manual of mental disorders, fourth edition(-text revision), **EET** Emotion Evaluation Test, **EF** executive functioning, **EUC** non-familial childhood-onset Epilepsy of Unknown Cause, **FRT** Facial Recognition Task, **GMDS** Griffiths Mental Developmental Scales, **GO4KIDDS** Great Outcomes for Kids Impacted by Severe Developmental Disabilities Brief Adaptive scale, **HC** healthy controls, **iASD** idiopathic autism spectrum disorder, **ICD** International Classification of Diseases, **I-MCH-FS** Italian version of the Montreal Children’s Hospital Feeding Scale, **Leiter-R** Leiter International Performance Scale-Revised, **M-CHAT** Modified Checklist for Autism in Toddlers-Revised, **MS2D** Memory Search 2D Objects, **NEPSY-II** NEuroPSYchological Assessment, 2^nd^ edition, **NF1** neurofibromatosis type 1, **NF1iv** neurofibromatosis type 1 caused by an intragenic variant in *NF1* **NF1md** neurofibromatosis type 1 caused by an *NF1* microdeletion, **NS** Noonan syndrome, **NS-LAH** Noonan-like syndrome with loose anagen hair, **NS-ML** Noonan syndrome with multiple lentigines, **ROA** Response Organization Arrows, **SCQ** Social Communication Questionnaire, **SDQ** Strengths and Difficulties Questionnaire, **SEARS-P** Social Emotional Assets and Resilience Scales-Parent, **SON** Snijders-Oomen Non-verbal intelligence test, **SP** Sensory Profile, **SPSS** Social Performance Survey Schedule, **SRS** Social Responsiveness Scale, **SSIS** Social Skills Improvement System, **SSRS** Social Skills Rating System, **TEA-Ch** Test of Everyday Attention for Children, **TOF** Test Observation Form, **TSC** Tuberous Sclerosis Complex, **US** unaffected siblings of RASopathy patients, **US-ASD** Unaffected siblings of individuals with ASD, **VABS-II** Vineland Adaptive Behaviour Scales-2^nd^ edition, **VADPRS** Vanderbilt ADHD Diagnostic Parent Rating Scale, **VRB** Visualization and Reasoning Battery, **WAIS(-R**) Wechsler Adult Intelligence Scale(-Revised), **WASI(-II/III)** Wechsler Abbreviated Scale of Intelligence(-2^nd^ edition/3^rd^ edition), **WISC(-III/IV/V)** Wechsler Intelligence Scale for Children(-3^rd^ edition/4^th^ edition/5^th^ edition), **WPPSI(-III/IV)** Wechsler Preschool and Primary Scale of Intelligence(-3^rd^ edition/ 4^th^ edition)

### TABLE S2: Rating of included cross-sectional studies according to the STROBE recommendations

|  | ^4^ | ^5^ | ^6^ | ^7^ | ^8^ | ^9^ | ^10^ | ^11^ | ^12^ | ^13^ | ^14^ | ^15^ | ^16^ | ^17^ | ^18^ | ^19^ | ^20^ | ^21^ | ^22^ | ^23^ | ^24^ | ^25^ | ^26^ | ^1^ | ^28^ | ^2^ | ^29^ | ^30^ | ^31^ | ^32^ | ^33^ | ^34^ | ^35^ | ^36^ | ^37^ | ^38^ | ^39^ | ^40^ | ^41^ | ^42^ |
| --- | --- | --- | --- | --- | --- | --- | --- | --- | --- | --- | --- | --- | --- | --- | --- | --- | --- | --- | --- | --- | --- | --- | --- | --- | --- | --- | --- | --- | --- | --- | --- | --- | --- | --- | --- | --- | --- | --- | --- | --- |
| 1a | - | + | + | - | + | - | - | + | - | - | - | - | - | - | - | - | - | - | - | - | - | NA | - | - | + | - | - | - | + | + | + | - | + | - | - | - | - | - | - | - |
| 1b | + | + | + | + | + | + | + | + | + | + | + | + | + | + | + | + | + | + | + | + | + | NA | + | NA | + | + | + | + | + | + | + | + | + | + | + | + | + | + | + | + |
| 2 | + | + | + | + | + | + | + | + | + | + | + | + | + | + | + | + | + | + | + | + | + | + | + | + | + | + | + | + | + | + | + | + | + | + | + | + | + | + | + | + |
| 3 | + | + | + | + | + | + | + | + | + | + | + | + | + | + | + | + | + | + | + | + | + | + | + | + | + | + | + | + | + | + | + | + | + | + | + | + | + | + | + | + |
| 4 | - | + | + | + | + | + | - | + | - | - | + | - | + | - | - | + | + | + | + | + | + | + | + | + | + | + | + | + | + | + | + | + | + | + | + | + | + | + | + | + |
| 5 | - | + | - | + | - | - | - | + | - | - | - | - | - | + | + | - | - | + | + | + | + | - | - | - | - | - | - | + | - | + | + | + | + | + | - | - | + | - | + | + |
| 6a | - | + | + | - | - | + | + | + | - | + | - | - | + | + | + | + | + | + | + | + | + | + | + | - | - | - | + | + | + | + | + | + | - | + | + | - | + | + | + | + |
| 6b | NA | NA | NA | NA | NA | NA | NA | NA | NA | NA | NA | NA | NA | NA | NA | NA | NA | NA | NA | NA | NA | NA | NA | NA | NA | NA | NA | NA | NA | + | NA | NA | NA | NA | NA | NA | NA | NA | NA | NA |
| 7 | + | + | + | + | + | + | + | + | + | + | + | + | + | + | + | + | + | + | + | + | + | + | + | + | + | + | + | + | + | + | + | + | + | + | + | + | + | + | + | + |
| 8 | + | + | + | + | + | + | + | + | + | + | + | + | + | + | + | + | + | + | - | + | + | + | + | + | + | + | + | + | + | + | + | + | + | + | + | + | + | + | + | + |
| 9 | - | + | + | - | - | + | - | - | - | - | - | - | - | + | - | - | - | - | - | + | + | - | - | - | + | - | + | + | - | + | + | - | - | + | - | + | + | + | + | + |
| 10 | - | - | + | - | - | - | - | + | + | + | - | + | + | + | - | + | - | - | + | + | + | + | - | + | + | - | + | + | - | + | + | - | - | - | - | - | + | + | - | - |
| 11 | + | + | + | + | + | + | + | + | + | + | + | + | + | + | + | + | + | + | + | + | + | + | + | + | + | + | + | + | + | + | + | + | + | + | + | + | + | + | + | + |
| 12a | + | + | + | + | + | + | + | + | - | + | + | + | + | + | + | + | + | + | + | + | - | + | + | - | + | + | + | + | + | + | + | + | + | + | + | + | + | + | NA | + |
| 12b | NA | NA | NA | + | + | + | NA | NA | - | NA | NA | + | + | NA | + | + | NA | + | + | NA | - | + | + | - | NA | NA | NA | NA | + | + | + | + | + | + | + | + | + | + | + | + |
| 12c | - | + | - | - | + | + | - | + | + | - | - | - | + | + | + | + | - | - | + | + | - | NA | - | + | - | + | - | - | - | + | - | - | + | - | + | + | NA | + | - | - |
| 12d | NA | + | NA | - | NA | - | - | NA | - | - | NA | NA | NA | - | - | - | - | - | NA | NA | NA | - | - | NA | - | NA | - | NA | + | + | - | NA | - | + | NA | NA | - | NA | NA | - |
| 12e | - | - | - | - | + | + | - | - | - | - | - | - | - | + | - | + | - | - | - | - | - | - | - | - | - | - | - | - | + | - | - | - | - | + | - | + | + | - | - | - |
| 13a | - | + | + | + | - | - | - | + | + | - | - | + | - | + | - | + | - | - | - | - | + | + | - | - | - | - | + | + | + | + | + | - | + | - | - | + | + | + | + | - |
| 13b | - | - | - | + | - | - | - | + | + | - | - | + | - | + | - | - | - | - | - | - | + | + | - | + | - | - | + | + | - | + | + | - | + | - | + | + | + | + | + | - |
| 13c | - | - | - | + | - | - | - | + | - | - | - | - | - | - | - | - | - | - | - | - | - | - | - | - | - | - | - | - | - | NA | + | - | + | - | - | + | + | - | - | - |
| 14a | + | + | + | + | + | + | + | + | - | + | + | + | + | + | + | + | + | + | + | + | - | + | + | - | + | + | + | + | + | + | + | + | + | + | + | + | + | + | + | + |
| 14b | - | + | + | + | + | + | + | + | + | + | - | - | + | + | + | + | + | + | + | + | + | NA | - | - | - | + | - | + | - | NA | + | - | + | - | + | + | NA | + | NA | - |
| 14c | NA | NA | NA | NA | NA | NA | NA | NA | NA | NA | NA | NA | NA | NA | NA | NA | NA | NA | NA | NA | NA | NA | NA | NA | NA | NA | NA | NA | - | + | NA | NA | NA | NA | NA | NA | NA | NA | NA | NA |
| 15 | - | + | + | + | + | + | + | + | + | + | + | + | + | + | + | + | + | + | + | + | + | + | - | - | + | + | + | + | + | + | + | + | + | + | + | + | + | + | + | + |
| 16a | + | + | + | + | + | + | + | + | + | + | + | + | + | + | + | + | + | + | + | - | + | + | + | + | + | + | + | + | + | + | + | + | + | + | + | + | + | + | + | + |
| 16b | NA | NA | + | + | + | + | + | + | + | + | NA | + | + | NA | + | + | NA | + | + | + | + | + | + | + | + | + | + | + | + | NA | + | + | + | NA | NA | + | + | + | + | + |
| 16c | NA | NA | NA | NA | NA | NA | NA | NA | NA | NA | NA | NA | NA | NA | NA | NA | NA | NA | NA | NA | NA | NA | NA | NA | NA | NA | NA | NA | NA | + | NA | NA | NA | NA | NA | NA | NA | NA | NA | NA |
| 17 | NA | + | + | + | + | + | NA | NA | + | + | NA | + | + | NA | + | + | NA | + | + | NA | + | + | + | + | NA | NA | NA | NA | + | + | + | + | + | + | + | + | + | + | + | + |
| 18 | + | + | + | + | + | + | + | + | + | + | + | + | + | + | + | + | + | + | + | + | + | + | + | + | + | + | + | + | + | + | + | + | + | + | + | + | + | + | + | + |
| 19 | - | + | + | + | + | - | + | + | + | + | + | + | + | + | + | + | + | + | + | + | + | - | + | + | - | + | + | + | + | + | + | + | + | + | + | + | + | + | + | + |
| 20 | + | + | + | + | + | + | - | + | + | + | + | + | + | + | + | + | + | + | + | + | + | - | + | + | + | + | + | + | + | + | + | + | + | + | + | + | + | + | + | + |
| 21 | - | + | + | + | + | + | + | + | + | + | - | + | + | + | + | + | + | + | + | + | + | - | + | + | + | + | + | + | + | + | + | - | - | + | + | + | + | + | + | + |
| 22 | + | + | + | + | - | + | + | + | + | - | + | + | - | + | + | + | + | + | + | + | + | + | + | + | + | + | + | + | + | + | + | - | + | + | + | + | - | + | + | + |

Rating of included cross-sectional studies according to the Strengthening the Reporting of Observational Studies in Epidemiology (STROBE) recommendations, checklist for cross-sectional studies. Study references are given in the top row, checklist items in the first column. NA: Not applicable, -: Recommendation not fulfilled, +: Recommendation fulfilled.
Recommendations are: 1a) Indicate the study’s design with a commonly used term in the title or the abstract; 1b) Provide in the abstract an informative and balanced summary of what was done and what was found; 2) Explain the scientific background and rationale for the investigation being reported; 3) State specific objectives, including any prespecified hypotheses; 4) Present key elements of study design early in the paper; 5) Describe the setting, locations, and relevant dates, including periods of recruitment, exposure, follow-up, and data collection; 6a) Give the eligibility criteria, and the sources and methods of selection of participants; 6b) Cohort study – For matched studies, give matching criteria and the number of controls per case; 7) Clearly define all outcomes, exposures, predictors, potential confounders, and effect modifiers. Give diagnostic criteria, if applicable; 8) For each variable of interest, give sources of data and details of methods of assessment (measurement). Describe comparability of assessment methods if there is more than one group; 9) Describe any efforts to address potential sources of bias; 10) Explain how the study size was arrived at; 11) Explain how quantitative variables were handled in the analyses. If applicable, describe which groupings were chosen and why; 12a) Describe all statistical methods, including those used to control for confounding; 12b) Describe any methods used to examine subgroups and interactions; 12c) Explain how missing data were addressed; 12d) If applicable, describe analytical methods taking account of sampling strategy; 12e) Describe any sensitivity analyses; 13a) Report numbers of individuals at each stage of study—eg numbers potentially eligible, examined for eligibility, confirmed eligible, included in the study, completing follow-up, and analysed; 13b) Give reasons for non-participation at each stage; 13c) Consider use of a flow diagram; 14a) Give characteristics of study participants (eg demographic, clinical, social) and information on exposures and potential confounders; 14b) Indicate number of participants with missing data for each variable of interest; 14c) Cohort study – Summarise follow-up time; 15) Cohort study – Report numbers of outcome events or summary measures over time; Cross-sectional study - Report numbers of outcome events or summary measures; 16a) Give unadjusted estimates and, if applicable, confounder-adjusted estimates and their precision (eg, 95% confidence interval). Make clear which confounders were adjusted for and why they were included; 16b) Report category boundaries when continuous variables were categorized; 16c) If relevant, consider translating estimates of relative risk into absolute risk for a meaningful time period; 17) Report other analyses done—eg analyses of subgroups and interactions, and sensitivity analyses; 18) Summarise key results with reference to study objectives; 19) Discuss limitations of the study, taking into account sources of potential bias or imprecision. Discuss both direction and magnitude of any potential bias; 20) Give a cautious overall interpretation of results considering objectives, limitations, multiplicity of analyses, results from similar studies, and other relevant evidence; 21) Discuss the generalisability (external validity) of the study results; 22) Give the source of funding and the role of the funders for the present study and, if applicable, for the original study on which the present article is based

TABLE S3: Outcome of included studies

| **Authors and year** | **ASD instruments** | **Main outcomes** |
| --- | --- | --- |
| Huijbregts et al., 2011^4^ | SRS | * NF1 SRS Mean Total T-score 59.4.  * SRS Mean Total T-score significantly higher in NF1 than HC, also after correction for total cognition. |
| ^†^Garg, Green, et al., 2013^5^ | SRS, ADI-R, ADOS-G | * 29.8% of NF1 meet criteria for ASD according to CPEA criteria, 27.7% broad ASD, 42.5% non-ASD.  * All ADI-R and ADOS subscales significantly different between NF1+ASD and NF1-nonASD (p<0.05). |
| Garg, Lehtonen et al., 2013^6^ | SRS | * NF1 Mean SRS Total T-score 63.05.  * 56.0% (parent) or 30.2% (teacher) Total T-score ≥60.  * 29.4% (parent) or 5.7% (teacher) Total T-score ≥76. |
| van Eeghen et al., 2013^7^ | SRS | * NF1 SRS Mean Total T-score 60, with unimodal distribution.  * 40% of NF1 Total T-score ≥60. * 18% of NF1 Total T-score ≥76. * NF1 SRS Total T-score and subdomain T-scores lower than iASD. * All disorders similar subdomain profile: Autistic mannerisms and Social cognition most impaired, Social awareness least impaired. |
| Walsh et al., 2013^8^ | SRS | * NF1 SRS Mean Total T-score 57.9.  * Moderate and severe deficits most common in subscale Autistic mannerism, least common in Social cognition and Social awareness. |
| Adviento et al., 2014^9^ | SCQ, SRS, ADOS, ADI-R | * 27% total RASopathy group, 54% CFC, 26% CS, 21% NS, 11% NF1, 85% iASD reach threshold SCQ.  * CFC SRS Mean Total T-score significantly higher than other RASopathies.  * RASopathies SRS Mean Total T-score significantly higher than US.  * Similar pattern of SRS subscale scores in RASopathy participants compared to iASD.  * ADOS and ADI-R only administered in fraction of participants, allowing no comparisons |
| Alfieri et al., 2014^10^ | SCQ, M-CHAT, DSM-IV criteria | * 64% in CFC, 44% in CS, 12% in NS reach threshold on SCQ or M-CHAT.  * CFC and CS reach threshold significantly more often than NS (p<0.01).  * Of those reaching threshold, 0% in NS, 71% in CFC and 25% in CS meet DSM-IV-criteria. |
| Tinker et al., 2014^11^ | M-CHAT, CAST | * Proportion screening positive on M-CHAT and CAST similar in NF1 and normative population.  * CAST mean and median score in NF1 significantly higher than normative population. |
| Constantino et al., 2015^12^ | SRS | * NF1 SRS Mean Total T-score 58.43  * Significant correlation between SRS Total T-score and ADHD symptoms |
| ^‡^Garg et al., 2015^13^ | SRS, ADOS-2 | * NF+ASD SRS Mean Total T-score 78.58.  * 67% of NF1+ASD clinically significant impairments on Autistic mannerisms.  * NF1+ASD no significantly different score on ADOS-2 SA as iASD norms, but several SA items significantly more impaired in the NF1+ASD group.  * RRB and SA item Eye contact less impaired in NF1+ASD compared iASD norms. |
| Loitfelder et al., 2015^14^ | SRS | * NF1 significantly higher scores on SRS Total T-score and all SRS subscales than HC.  * After correction for cognition and executive function, only Total score, Social motivation, and Autistic mannerisms significantly higher in NF1. |
| Niemczyk et al., 2015^15^ | DBC-P, DBC-A | * NS Children: 35.3% above cut-off DBC-P.  * NS Adults: 10% over cut-off DBC-A. |
| Plasschaert et al., 2015^16^ | CSBQ, SRS | * NF1 SRS Mean Total T-score 60.76.  * SRS subscales Social cognition in NF1 most severely affected, but all subscales in NF1 significantly higher than norms from general population.  * All CSBQ subscales more impaired than norms from general population. |
| Bilder et al., 2016^17^ | DSM-IV-TR | * ASD+NF1 children significantly less likely to meet DSM-IV-TR criterion 1a (difficulty using or understanding non-verbal communication) than ASD-NF1 (58 vs. 84%; p = 0.001) * Age at first ASD diagnosis not significantly different between ASD+NF1 and ASD-NF1 |
| ^§^Garg et al., 2016^18^ | SRS, ADI-R, ADOS | * NF1 SRS Total Raw Score in males 89.50, in females 69.33 (p < 0.001), and all subscale Raw Scores higher in males than females * NF1 SRS Total T-score in males 72.17, in females 67.00 (p = 0.025)  * NF1+ASD SRS Mean T-score in males 84.35, in females 85.33 (p = 0.797)  * Male to female ratio meeting ASD criteria 2.68:1  * NF1+ASD males more social communication deficits than females, but similar RRB on ADOS and ADI-R. |
| Morris et al., 2016^19¶^ | SRS | * NF1 SRS Mean T-score 58.21.  * NF1+ASD SRS Mean T-score 78.59 (n = 79). |
| Plasschaert et al., 2016^20^ | SRS | * NF1 SRS Social problems and Mannerisms subscale scores significantly higher compared to HC, but lower compared to iASD (p < 0.001). |
| Garg et al., 2017^21^ | ADOS, ADI-R | * ASD in 30% of NS and 89% of CFC.  * ADOS and ADI-R subscale scores not different in NS and CFC. |
| Schwartz et al., 2017^22^ | M-CHAT, SCQ | * Younger than 7y: 71% in CS met liberal cut-off M-CHAT.  * Older than 7y: 0% in CS met cut-off SCQ. |
| Eijk et al., 2018^23^ | SRS, ADOS-G, ADOS-2, DSM-IV | * NF1 SRS Mean Total T-score 54.7  * 10.9% of NF1 clinical DSM-IV ASD diagnosis * 18.8% of NF1 ASD classification on ADOS. |
| Hirabaru et al., 2018^24^ | SRS | * 20.3% of NF1 in severe clinical range on SRS. * 14.7% of NF1 in mild-moderate clinical range on SRS |
| °Morris et al., 2018^25^ | SRS | * Individuals with variants within the 5’-end of the NF1 gene significantly lower QAT scores than those with variants within the 3’-end (p = 0.03). |
| Pierpont et al., 2018^26^ | SSIS | * 5% of NS and 10% of NF1 previous ASD diagnosis  * 41% of NS, 44% of NF1 and 13% of US scored outside of normal SSIS limits, with 15% of NS and 5% of NF1 in severe social impairment range.  * NS and NF1 similar social skills, and significantly lower than US. |
| Stivaros et al., 2018^27^ | ADI-R, ADOS-2, SRS | * NF1+ASD SRS Mean Total T-score 83.00. |
| ^#^Young et al., 2018^1^ | SCQ, SRS | * No correlation between age and SRS score  * Significantly higher ASD rate according to SCQ in older (>4 years) group. |
| Kehrer-Sawatzki et al., 2020^2^ | SRS | * SRS Mean Total T-score in NF1md 64.6, significantly higher than published mean scores for general NF1  * 70.8% of NF1md have SRS Total T-score ≥60 and <76, significantly more often than published ratios for general NF1 |
| Payne et al., 2020^28^ | SRS, SSIS-RS | * NF1 SRS Mean Total T-score 59.9  * 16.4% of NF1 SRS Total T-score in the severe range and 18.8% in mild-moderate range  * 8.2% of NF1 SSIS-RS total > 75 |
| ^£^Geoffray et al., 2021^29^ | ADOS-2, ADI-R | * ADOS SA subscale score not different in NS+ASD, CFC+ASD and NF1+ASD (p = 0.078).  * Lower ADOS RRB subscale in NF1+ASD compared to CFC+ASD and NS+ASD (p = 0.009).  * No significant differences between NF1+ASD, NS+ASD and CFC+ASD on ADI-R scores. |
| Morotti et al., 2021^30^ | SRS, SCQ, RBS-R | * NF1 SRS Mean Total T-score 55.8.  * NF1 SRS and SCQ scores significantly lower in iASD, but significantly higher than US-ASD (p < 0.001)  * NF1 RBS-R scores significantly lower than in iASD (p < 0.001) |
| Glad et al., 2021^31^ | SSRS, SSIS | * No difference between NF1 males and females in Social skills  * Social skills <1SD below the normative mean in 32% in early childhood and 24% at school age  * Social skills <2SD below the normative mean in 4% in early childhood and 8% at school age  * No differences in social skills by sex  * Early childhood social skills and school age social skills not different  * Early childhood social skills positively correlated with age, but school age social skills not  * Social skills at 5-6 years positively correlated with social skills at school age  * Social skills 3 years < 6 years  * Hyperactivity and inattention in early childhood negatively correlated with social skills in early childhood  * Both inattention in early childhood and hyperactivity and inattention at school age negatively corelated with social skills at school age  * Cognitive functioning not correlated with social skills |
| Kenborg et al., 2021^32^ | ICD code in DNPR | * Only significantly higher hazard ratio for ASD-related hospital contact in NF1 compared to general population in age group 0-7 years  * Hazard ratio for ASD-related hospital contact not different between males with NF1 and females with NF1 |
| ^¥^Chisholm et al., 2022^33^ | SRS, ADOS, ADI-R | * Mean NF1 SRS Total T-score in entire sample 62.4  * 33.8% of sample with SRS Total T-score ≥ 60 meets ADI-R algorithm cut-off  * 63.1% of sample with SRS Total T-score ≥ 60 meets ADOS-2 algorithm cut-off  * ADI-R and ADOS items most endorsed for peer relationships, reciprocal conversation, eye contact, social smiling, abnormal pointing, social overtures and responses and reciprocal verbal and nonverbal social communication, difficulty with minor changes, compulsions and rituals, sensory sensitivities, circumscribed interests. Minimal motor stereotypies  * Weak correlations between hyperactivity/impulsivity and ADI-R scores. No correlations with ADOS-2 scores  * Weak to moderate correlations between SRS Total T-scores and hyperactivity/impulsivity and inattention. |
| Cohen et al., 2022^34^ | SCQ | * 10% of NS participants met SCQ cut-off. All had an ADHD diagnosis. |
| Foy et al., 2022^35^ | SEARS-P | * Previous ASD diagnoses: NF1 16%, NS 10%, CS 6%, CFC 23%  * Social competence and empathy in RASopathy > iASD  * Higher difference between SEARS-P subscales favouring empathy in RASopathy compared to iASD, also consistent across the 4 syndromes  * Higher Hyperactivity/Inattention predictive of lower social competence and lower empathy  * Rasopathy+ASD similar risk of severe deficits in social competence as iASD, but less likely to have severe deficits in empathy |
| ^¥^Haebich et al., 2022^36^ | SSIS | * In NF1 significant, but weak correlation social skills with FSIQ and sex, but not with age  * In NF1 significant moderate to strong intercorrelations between social skills, ADHD symptoms an executive functions  * ADHD symptoms mediate the relationship between executive functions and social skills |
| Lalancette et al., 2022^37^ | SRS | * Mean NF1 SRS Total T-score 58.50 |
| Lubbers et al., 2022^38^ | SRS, ADOS | * Mean NF1 SRS Total T-score 55.7  * 29.0% of NF1 participants SRS Total T-score ≥ 60  * 20.0% of NF1 participants ADOS ASD classification  * SRS Total, Social communication and interaction and Restricted and repetitive behavior T scores in NF1+ASD lower than in iASD  * Except for Social cognition, SRS subscale scores lower in NF1+ASD compared to iASD, but similar profile  * ADOS Total, Social affect and Restricted and repetitive behavior CSS in NF1+ASD not different from iASD  * ADOS weighted subscale scores in NF1+ASD and iASD not different, and similar profile |
| ^¥^Chisholm et al., 2023^39^ | SRS, ADOS, ADI-R | * NF1 males higher impairment on ADI-R Social and Communication domains, driven by items on nonverbal behaviours, shared enjoyment, socioemotional reciprocity, make-believe and social imitative play and stereotyped, repetitive or idiosyncratic speech  * NF1 males higher impairment on ADOS Social affect subscale  * Higher proportion of NF1 males exceeding ADI-R Social and Communication cut-offs as well as ADOS autism spectrum cut-off  * Twice as many NF1 males exceed all three ADI-R cut-offs, but not statistically significant  * No sex differences on the ADI-R and ADOS RRB domain  * Significant differences between lifetime and current scores on ADI-R suggest abatement of autistic behaviours in males for Social and Communication, and in both sexes for RRB. However, high variability in individual participants |
| Naylor et al., 2023^40^ | KSADS, SRS | * No NS participants met DSM-5 criterion A for ASD according to KSADS, 9.5% met criterion B  * Parents frequently reported deficits in developing and maintaining friendships, incessant pursuit of others, insistence on sameness and hyper- or hyporeactivity to sensory input  * NS Mean SRS Total T-score 65.72  * NS Mean SRS Total T-score ≥ 60 in 52.4% and Total T-score >75 in 26.2% |
| Onesimo et al., 2023^41^ | SP | * 45% of CFC participants had scores in the atypical range for modality Oral |
| ^¥^Pride et al., 2023^42^ | SRS, SP, SSIS | * Mean NF1 SRS Total T-score 61.9  * Higher scores and higher proportions of NF1 participants exceeding the normal range of SP Sensory seeking, Sensory avoidance, Hypersensitivity and Low registration  * NF1 higher scores on modalities Auditory, Touch, Movement, Body position and Oral than HC  * NF1 participants more likely to be impacted on multiple quadrants and modalities than HC  * NF1 group similar to published ASD data on quadrants Registration and Seeking, but lower on Sensitivity and Avoidance  * SP quadrant scores not associated with sex or age; but positively with SRS total T-scores, Hyperactivity/impulsivity and Inattention. Weak negative relationship between FSIQ and Sensory registration and Sensory seeking  * Negative correlation between quadrant scores and ABAS, SSIS scores |

^†^ Examined a subset of participants from Garg, Lehtonen et al., 2013, stratified according to SRS T-scores ^‡^ Pooled NF1+ASD data from the samples of Garg, Green, et al., 2013, Plasschaert et al., 2015, and Stivaros et al., 2018. ^§^ Pooled data from Garg, Green et al., 2013, and Stivaros et al., 2018. ^¶^ Pooled original data with those from Garg, Lehtonen, et al., 2013, Walsh et al., 2013, Adviento et al., 2014, Constantino et al., 2015, and Plasschaert et al., 2015. ° Analyzed a subset of data from Constantino et al., 2015. ^#^ Pooled original data with those from Adviento et al., 2014 ^£^ Pooled a subset of data of NF1+ASD, CFC+ASD and CS+ASD from Garg, Green et al., 2013, Garg et al., 2017, and Stivaros et al., 2018. ^¥^ Chisholm et al., 2022, Haebich et al., 2022, Chisholm et al., 2023 and Pride et al., 2023 report data from overlapping samples from the same overarching study.

**ADHD-RS** Attention Deficit Hyperactivity Disorder–Rating Scale, **ADI-R** Autism Diagnostic Interview-Revised, **ADOS** Autism Diagnostic Observation Schedule, **CAST** Childhood Autism Spectrum Test, **CFC** cardio-facio-cutaneous syndrome, **CS** Costello syndrome, **CSBQ** Children’s Social Behaviour Questionnaire, **DBC-A** Developmental Behaviour Checklist-Adult, **DBC-P** Developmental Behaviour Checklist-Parent, **DSM-IV-TR** Diagnostic and Statistical Manual of mental disorders, fourth edition-Text Revision, **EUC** non-familial childhood-onset Epilepsy of Unknown Cause, **HC** healthy controls, **iASD** idiopathic ASD, **M-CHAT** Modified Checklist for Autism in Toddlers, **NF1** neurofibromatosis type 1, **NF1md** neurofibromatosis type 1 caused by an *NF1* microdeletion, **NS** Noonan syndrome, **RRB** ADOS Restricted and Repetitive Behavior subscale, **SA** ADOS Social Affect subscale, **SCQ** Social Communication Questionnaire, **SP** Sensory Profile, **SPSS** Social Performance Survey Schedule, **SRS** Social Responsiveness Scale, **SSIS** Social Skills Improvement System, **TSC** Tuberous Sclerosis Complex, **US** unaffected siblings of RASopathy patients, **US-ASD** Unaffected siblings of individuals with ASD

**Supplement 3: Estimation of the amount of unique participants across the included studies**

While processing the data, in some cases it became unambiguously that there was sample overlap between the included articles. In other cases it was highly probable, and in still other cases there was no clear evidence in that direction, but it could not be completely ruled out. We thought it relevant to provide an estimate of the number of unique participants, and not simply a sum of the sample sizes of the articles. Our estimate of the number of unique participants is not necessarily the exact number that would be found if calculations could be done based on completely transparent, unambiguous information on the overlap between the different samples. However the information in the articles does not allow this accuracy. At the same time, there is no doubt that our estimate is much closer to the exact amount of unique participants than the sum of the numbers of the different articles, which would constitute a clear overestimation.

We determined for every RASopathy whether there was substantial sample overlap based on: 1) mention in the articles themselves (e.g. reference to another article for participant recruitment methods or source of the data, or reference to the same previously published research protocol) or 2) same type of data (e.g. SRS scores) from the same authors from the same center with overlap in the recruitment period. In case of overlap, we favored the largest sample size to be included in our count (e.g. Lubbers et al., 2022 n=279 instead of Eijk et al., 2018 n=128).

NF1

We reached the estimate of 2601 unique NF1participants by adding up the participants of the following studies:

| Article | N |
| --- | --- |
| Huijbregts et al., 2011^4^ | 30 |
| Van Eeghen et al., 2013^7^ | 50 |
| Tinker et al., 2014^11^ | 67 |
| Bilder et al., 2016^17^ | 22 |
| Garg et al., 2016 (only the SANTA study subsample)^18^ | 85 |
| Morris et al., 2016^19^ | 531 |
| Hirabaru et al., 2018^24^ | 143 |
| Pierpont et al., 2018^26^ | 39 |
| Kehrer-Sawatzki et al., 2020^2^ | 60 |
| Morotti et al., 2021^30^ | 45 |
| Glad et al., 2021^31^ | 65 |
| Kenborg et al., 2021^32^ | 905 |
| Cohen et al., 2022^34^ | 30 |
| Foy et al., 2022^35^ | 70 |
| Lubbers et al., 2022^38^ | 279 |
| Lalancette et al., 2022^37^ | 28 |
| Pride et al., 2023^42^ | 152 |

We did not include the following articles in the count:

| Article | N | Reason |
| --- | --- | --- |
| Garg, Green et al., 2013^5^ | 47 | Overlap with Morris et al., 2016^19^ |
| Garg, Lehtonen et al., 2013^6^ | 109 | Overlap with Morris et al., 2016^19^ |
| Walsh et al., 2013^8^ | 66 | Overlap with Morris et al., 2016^19^ |
| Adviento et al., 2014^9^ | 81 | Overlap with Morris et al., 2016^19^ |
| Constantino et al., 2015^12^ | 103 | Overlap with Morris et al., 2016^19^ |
| Garg et al., 2015^13^ | 36 | Overlap with Morris et al., 2016^19^ |
| Loitfelder et al., 2015^14^ | 14 | Overlap with Huijbregts et al., 2011^4^ |
| Plasschaert et al., 2015^16^ | 82 | Overlap with Morris et al., 2016^19^ |
| Garg et al., 2016^18^ | 194 | Overlap with Morris et al., 2016^19^ (except the SANTA study subsample) |
| Eijk et al., 2018^23^ | 128 | Overlap with Lubbers et al., 2022^38^ |
| Morris et al., 2018^25^ | 63 | Overlap with Morris et al., 2016^19^ |
| Stivaros et al., 2020^27^ | 28 | Overlap with Garg et al., 2016^18^ |
| Payne et al., 2020^28^ | 122 | Overlap with Pride et al., 2023^42^ and Glad et al., 2021^31^ |
| Geoffray et al., 2021^29^ | 48 | Overlap with Garg et al., 2016^18^ |
| Chisholm et al., 2022^33^ | 68 | Overlap with Pride et al., 2023^42^ |
| Haebich et al., 2022^36^ | 136 | Overlap with Pride et al., 2023^42^ |
| Chisholm et al., 2023^39^ | 62 | Overlap with Pride et al., 2023^42^ |

NS

We reached the estimate of 313 unique NS participants by adding up the participants of the following studies:

| Article | N |
| --- | --- |
| Adviento et al., 2014^9^ | 52 |
| Alfieri et al., 2014^10^ | 38 |
| Niemczyk et al., 2015^15^ | 29 |
| Garg et al., 2017^21^ | 40 |
| Pierpont et al., 2018^26^ | 39 |
| Foy et al., 2022^35^ | 70 |
| Naylor et al., 2023^40^ | 45 |

We did not include the following article in the count:

| Article | N | Reason |
| --- | --- | --- |
| Geoffray et al., 2021^29^ | 11 | Overlap with Garg et al., 2017^21^ |

CS

We reached the estimate of 101 unique CS participants by adding up the participants of the following studies:

| Article | N |
| --- | --- |
| Alfieri et al., 2014^10^ | 11 |
| Schwartz et al., 2017^22^ | 14 |
| Young et al., 2018^1^ | 61* |
| Foy et al., 2022^35^ | 15 |

* Young et al. did not mention an exact total number of included participants, but only mentioned that they added new data to the existing data from Adviento et al., 2014 to reach 53 participants with complete SCQ data, and 47 participants with complete SRS data. Adviento et al. reported that in their sample of 44 CS participants they had 43 participants with SCQ data and 40 participants with SRS data. This in turn implies that Young et al. had to include SCQ data from 10 more participants (53-43=10) and SRS data from seven more participants (47–40=7). In the most extreme situation, these 10 participants with additional SCQ data and seven participants with additional SRS data do not overlap at all. This means that Young et al. have, at most, included 17 additional participants (10+7=17) compared to the total sample of Adviento et al. We can conclude that the maximum sample size of Young et al. is 61 (44+17). In case of maximum overlap between the participants who provided new SRS and SCQ data, data from 10 additional participants had to be included. Furthermore, the possibility exists that one of the participants who provided new SCQ data is the only participant from the dataset of Adviento et al. for whom SCQ data were absent. Thus, the minimum sample size of Young et al. is 53 (43+10). We decided to use the upper limit (61) in our calculations.

We did not include the following article in the count:

| Article | N | Reason |
| --- | --- | --- |
| Adviento et al., 2014^9^ | 44 | Overlap with Young et al., 2018^1^ |

CFC

We reached the estimate of 126 CFC participants by adding up the participants of the following studies:

| Article | N |
| --- | --- |
| Adviento et al., 2014^9^ | 54 |
| Alfieri et al., 2014^10^ | 11 |
| Garg et al., 2017^21^ | 9 |
| Foy et al., 2022^35^ | 25 |
| Onesimo et al., 2023^41^ | 27 |

Because we had no clear indications of sample overlap between the articles concerning CFC participants, all articles were included in the count.

**Supplement 4: supplementary figures**

Supplementary Figure 1: Mean SRS Total T-scores across RASopathies and studies


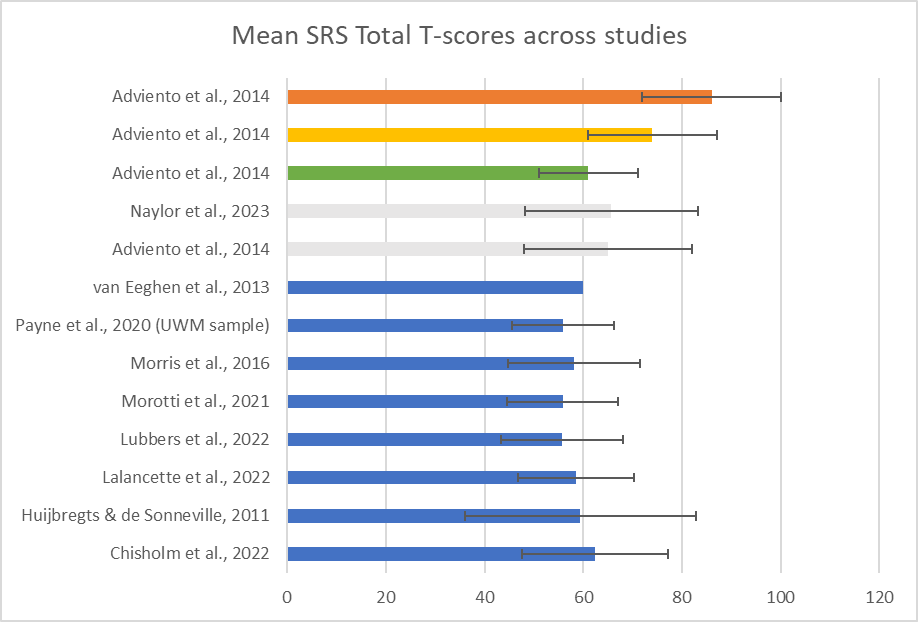


Figure S1: Mean SRS Total T-scores across studies. Orange = iASD, Yellow= CFC; Green = CS, Grey = NS; Blue = NF1. Error bars represent standard deviations. Note that van Eeghen et al. 2013 did not mention standard deviations.

**Supplementary references**

1. Young O, Perati S, Weiss LA, Rauen KA. Age and ASD symptoms in Costello syndrome. *Am J Med Genet A*. 2018;176(4):1027-1028. doi:10.1002/ajmg.a.38641

2. Kehrer-Sawatzki H, Kluwe L, Salamon J, et al. Clinical characterization of children and adolescents with NF1 microdeletions. *Child’s Nervous System*. 2020;36(10):2297-2310. doi:10.1007/s00381-020-04717-0

3. Pride NA, Crawford H, Payne JM, North KN. Social functioning in adults with neurofibromatosis type 1. *Res Dev Disabil*. 2013;34(10):3393-3399. doi:10.1016/j.ridd.2013.07.011

4. Huijbregts SCJ, de Sonneville LMJ. Does cognitive impairment explain behavioral and social problems of children with neurofibromatosis type 1? *Behav Genet*. 2011;41(3):430-436. doi:10.1007/s10519-010-9430-5

5. Garg S, Green J, Leadbitter K, et al. Neurofibromatosis Type 1 and Autism Spectrum Disorder. *Pediatrics*. 2013;132(6):e1642-8. Accessed September 14, 2018. www.aappublications.org/news

6. Garg S, Lehtonen A, Huson SM, et al. Autism and other psychiatric comorbidity in neurofibromatosis type 1: Evidence from a population-based study. *Dev Med Child Neurol*. 2013;55(2):139-145. doi:10.1111/DMCN.12043

7. van Eeghen AM, Pulsifer MB, Merker VL, et al. Understanding relationships between autism, intelligence, and epilepsy: A cross-disorder approach. *Dev Med Child Neurol*. 2013;55(2):146-153. doi:10.1111/DMCN.12044

8. Walsh KS, Vélez JI, Kardel PG, et al. Symptomatology of autism spectrum disorder in a population with neurofibromatosis type 1. *Dev Med Child Neurol*. 2013;55(2):131-138. doi:10.1111/dmcn.12038

9. Adviento B, Corbin IL, Widjaja F, et al. Autism traits in the RASopathies. *J Med Genet*. 2014;51(1):10-20. doi:10.1136/jmedgenet-2013-101951

10. Alfieri P, Piccini G, Caciolo C, et al. Behavioral profile in RASopathies. *Am J Med Genet A*. 2014;164(4):934-942. doi:10.1002/ajmg.a.36374

11. Tinker J, Carbone PS, Viskochil D, Mathiesen A, Ma KN, Stevenson DA. Screening children with neurofibromatosis type 1 for autism spectrum disorder. *Am J Med Genet A*. 2014;164A(7):1706-1712. doi:10.1002/ajmg.a.36549

12. Constantino JN, Zhang Y, Holzhauer K, et al. Distribution and within-family specificity of quantitative autistic traits in patients with neurofibromatosis type I. *J Pediatr*. 2015;167(3):621-26.e1. doi:10.1016/j.jpeds.2015.04.075

13. Garg S, Plasschaert E, Descheemaeker MJ, et al. Autism spectrum disorder profile in neurofibromatosis type I. *J Autism Dev Disord*. 2015;45(6):1649-1657. doi:10.1007/s10803-014-2321-5

14. Loitfelder M, Huijbregts SCJ, Veer IM, et al. Functional Connectivity Changes and Executive and Social Problems in Neurofibromatosis Type I. *Brain Connect*. 2015;5(5):320. doi:10.1089/BRAIN.2014.0334

15. Niemczyk J, Equit M, Borggrefe-Moussavian S, Curfs L, von Gontard A. Incontinence in persons with Noonan Syndrome. *J Pediatr Urol*. 2015;11(4):201.e1-201.e5. doi:10.1016/J.JPUROL.2015.06.002

16. Plasschaert E, Descheemaeker MJ, Van Eylen L, Noens I, Steyaert J, Legius E. Prevalence of autism spectrum disorder symptoms in children with neurofibromatosis type 1. *Am J Med Genet B Neuropsychiatr Genet*. 2015;168B(1):72-80. doi:10.1002/ajmg.b.32280

17. Bilder DA, Bakian A V., Stevenson DA, et al. Brief report: the prevalence of neurofibromatosis type 1 among children with autism spectrum disorder identified by the autism and developmental disabilities monitoring network. *J Autism Dev Disord*. 2016;46(10):3369-3376. doi:10.1007/s10803-016-2877-3

18. Garg S, Heuvelman H, Huson S, Tobin H, Green J, Northern UK NF1 Research Network. Sex bias in autism spectrum disorder in neurofibromatosis type 1. *J Neurodev Disord*. 2016;8:26. doi:10.1186/s11689-016-9159-4

19. Morris SM, Acosta MT, Garg S, et al. Disease burden and symptom structure of autism in neurofibromatosis type 1. A study of the international NF1-ASD consortium team (INFACT). *JAMA Psychiatry*. 2016;73(12):1276-1284. doi:10.1001/jamapsychiatry.2016.2600

20. Plasschaert E, Van Eylen L, Descheemaeker MJ, Noens I, Legius E, Steyaert J. Executive functioning deficits in children with neurofibromatosis type 1: the influence of intellectual and social functioning. *Am J Med Genet B Neuropsychiatr Genet*. 2016;171B(3):348-362. doi:10.1002/ajmg.b.32414

21. Garg S, Brooks A, Burns A, et al. Autism spectrum disorder and other neurobehavioural comorbidities in rare disorders of the Ras/MAPK pathway. *Dev Med Child Neurol*. 2017;59(5):544-549. doi:10.1111/DMCN.13394

22. Schwartz DD, Katzenstein JM, Highley EJ, et al. Age-related differences in prevalence of autism spectrum disorder symptoms in children and adolescents with Costello syndrome. *Am J Med Genet A*. 2017;173(5):1294-1300. doi:10.1002/AJMG.A.38174

23. Eijk S, Mous SE, Dieleman GC, et al. Autism spectrum disorder in an unselected cohort of children with neurofibromatosis type 1 (NF1). *J Autism Dev Disord*. 2018;48(7):2278-2285. doi:10.1007/s10803-018-3478-0

24. Hirabaru K, Matsuo M. Neurological comorbidity in children with neurofibromatosis type 1. *Pediatr Int*. 2018;60(1):70-75. doi:10.1111/ped.13388

25. Morris SM, Gutmann DH. A genotype–phenotype correlation for quantitative autistic trait burden in neurofibromatosis 1. *Neurology*. 2018;90(8):377-379. doi:10.1212/WNL.0000000000005000

26. Pierpont EI, Hudock RL, Foy AM, et al. Social skills in children with RASopathies: a comparison of Noonan syndrome and neurofibromatosis type 1. *J Neurodev Disord*. 2018;10(1):21. doi:10.1186/s11689-018-9239-8

27. Stivaros S, Garg S, Tziraki M, et al. Randomised controlled trial of simvastatin treatment for autism in young children with neurofibromatosis type 1 (SANTA). *Mol Autism*. 2018;9:12. doi:10.1186/S13229-018-0190-Z/TABLES/1

28. Payne JM, Walsh KS, Pride NA, et al. Social skills and autism spectrum disorder symptoms in children with neurofibromatosis type 1: evidence for clinical trial outcomes. *Dev Med Child Neurol*. 2020;62(7):813-819. doi:10.1111/DMCN.14517/ABSTRACT

29. Geoffray MM, Falissard B, Green J, et al. Autism Spectrum Disorder Symptom Profile Across the RASopathies. *Front Psychiatry*. 2021;11:585700. doi:10.3389/FPSYT.2020.585700/BIBTEX

30. Morotti H, Mastel S, Keller K, et al. Autism and attention-deficit/hyperactivity disorders and symptoms in children with neurofibromatosis type 1. *Dev Med Child Neurol*. 2021;63(2):226-232. doi:10.1111/dmcn.14558

31. Glad DM, Casnar CL, Yund BD, Lee K, Klein-Tasman BP. Parent-Reported Social Skills in Children with Neurofibromatosis Type 1: Longitudinal Patterns and Relations with Attention and Cognitive Functioning. *Journal of Developmental and Behavioral Pediatrics*. 2021;42(8):656-665. doi:10.1097/DBP.0000000000000939

32. Kenborg L, Andersen EW, Duun-Henriksen AK, et al. Psychiatric disorders in individuals with neurofibromatosis 1 in Denmark: A nationwide register-based cohort study. *Am J Med Genet A*. 2021;185(12):3706-3716. doi:10.1002/ajmg.a.62436

33. Chisholm AK, Haebich KM, Pride NA, et al. Delineating the autistic phenotype in children with neurofibromatosis type 1. *Mol Autism*. 2022;13(1):3. doi:10.1186/s13229-021-00481-3

34. Cohen R, Halevi A, Aharoni S, Aronson B, Diamond G. Impairments in communication and social interaction in children with neurofibromatosis type 1: Characteristics and role of ADHD and language delay. *Appl Neuropsychol Child*. 2022;11(3):220-225. doi:10.1080/21622965.2020.1780924

35. Foy AMH, Hudock RL, Shanley R, Pierpont EI. Social behavior in RASopathies and idiopathic autism. *J Neurodev Disord*. 2022;14(1):5. doi:10.1186/s11689-021-09414-w

36. Haebich KM, Dao DP, Pride NA, et al. The mediating role of ADHD symptoms between executive function and social skills in children with neurofibromatosis type 1. *Child Neuropsychology*. 2022;28(3):318-336. doi:10.1080/09297049.2021.1976129

37. Lalancette E, Charlebois-Poirier AR, Agbogba K, et al. Steady-state visual evoked potentials in children with neurofibromatosis type 1: associations with behavioral rating scales and impact of psychostimulant medication. *J Neurodev Disord*. 2022;14(1):42. doi:10.1186/s11689-022-09452-y

38. Lubbers K, Stijl EM, Dierckx B, et al. Autism Symptoms in Children and Young Adults With Fragile X Syndrome, Angelman Syndrome, Tuberous Sclerosis Complex, and Neurofibromatosis Type 1: A Cross-Syndrome Comparison. *Front Psychiatry*. 2022;13:852208. doi:10.3389/fpsyt.2022.852208

39. Chisholm AK, Lami F, Haebich KM, et al. Sex- and age-related differences in autistic behaviours in children with neurofibromatosis type 1. *J Autism Dev Disord*. 2023;53(7):2835-2850. doi:10.1007/s10803-022-05571-6

40. Naylor PE, Bruno JL, Shrestha SB, et al. Neuropsychiatric phenotypes in children with Noonan syndrome. *Dev Med Child Neurol*. 2023;65(11):1520-1529. doi:10.1111/dmcn.15627

41. Onesimo R, Sforza E, Giorgio V, et al. The “FEEDS (FEeding Eating Deglutition Skills)” over Time Study in Cardiofaciocutaneous Syndrome. *Genes (Basel)*. 2023;14(7):1338. doi:10.3390/genes14071338

42. Pride NA, Haebich KM, Walsh KS, et al. Sensory Processing in Children and Adolescents with Neurofibromatosis Type 1. *Cancers (Basel)*. 2023;15(14):3612. doi:10.3390/cancers15143612
